# An investigation of the genus *Mesacanthus* (Chordata: Acanthodii) from the Orcadian Basin and Midland Valley areas of Northern and Central Scotland using traditional morphometrics

**DOI:** 10.7717/peerj.1331

**Published:** 2015-10-29

**Authors:** Matthew G. Baron

**Affiliations:** Department of Earth Science, University of Cambridge, Cambridge, United Kingdom; Earth Sciences Department, Natural History Museum, London, United Kingdom

**Keywords:** Acanthodii, Morphometrics, *Mesacanthus*, Orcadian, Scotland

## Abstract

*Mesacanthus* is a common and speciose genus of acanthodian fish from Lower Old Red Sandstone and Middle Old Red Sandstone assemblages (representing the Lower Devonian and Middle Devonian respectively) and is well represented in many palaeoichthyology collections in the UK. Based upon descriptions given during the 19th century, specimens of the genus *Mesacanthus* from the Orcadian Basin and Midland Valley areas of Northern and Central Scotland have historically been referred to a number of different species; of these, the most frequently discussed in the literature are *M. mitchelli*, *M. peachi* and *M. pusillus*. In order to test the validity of these three species, traditional morphometric analyses were carried out on over 100 specimens of *Mesacanthus*, from both the Lower Devonian and the Middle Devonian, that cover the full range of known localities for these taxa in Northern and Central Scotland. Based upon morphological and morphometric comparisons, this investigation has found that at least two species of *Mesacanthus* are valid (*M. mitchelli* and *M. pusillus*) as specimens from the Lower Devonian and Middle Devonian have been shown to differ significantly in a number of important ways. However, no evidence has been found for the validity of the second and distinct Middle Devonian species, *M. peachi*.

## Introduction

Acanthodians (‘spiny sharks’) are important early representatives of Gnathostomata (Gegenbaur, 1874) and have been central in the debate on the origin of modern gnathostomes (Davis, Finarelli & Coates, 2012; Zhu et al., 2013). The most recent analyses have recovered acanthodians as a paraphyletic assemblage within the chondrichthyan total group (Dupret et al., 2014; Brazeau & Friedman, 2015; Giles, Friedman & Brazeau, 2015). Acanthodians have a global distribution and are generally characterised by the presence of long fin spines along the length of their body (Agassiz, 1833–1843; Agassiz, 1844–1845; Traquair, 1898; Miles, 1966; Denison, 1979). The genus *Mesacanthus* (Traquair, 1888) is found in the sedimentary rocks of the Orcadian Basin and Midland Valley areas of Northern and Central Scotland, in both the Lower Old Red Sandstone and the Middle Old Red Sandstone, which correspond to the Lower Devonian and Middle Devonian respectively. The sedimentary systems that they are found in are interpreted as being the remnants of extensive lake environments, which, despite fluctuations in their size and shape through time, remained a relatively permanent feature of the landscape of this region for millions of years (Trewin & Davidson, 1995; Trewin & Davidson, 1999; Newman & Trewin, 2008). The faunal composition markedly changed between the Lower and Middle Devonian, but the genus *Mesacanthus* is present in both of these stages and is quite common, representing up to 48 percent of the total number of specimens found in early Devonian formations (Trewin & Davidson, 1995).

Traquair (1888) erected the genus *Mesacanthus* having recognised that some of the species of *Acanthodes* (Agassiz, 1833–1843) that had been previously described by Agassiz (1844–1845) and Egerton (1861) were distinct enough from other species of *Acanthodes* to warrant their placement in a separate genus. He went on to state that *Mesacanthus* could be diagnosed and differentiated from other genera of acanthodians by the presence of a pair of prepelvics (intermediate spines between the pectoral and pelvic spines). These spines are small and delicate structures and are not always well preserved, making this particular character a difficult one to use on its own in diagnosing specimens. Prior to this work, Agassiz (1844–1845) had given a more detailed description of *Mesacanthus pusillus* (Agassiz, 1844–1845: *Acanthodes pusillus*) in which he stated that the species is extremely small, has short anal spines, triangular scales with raised keels on their top edge and a distinct tail in which the upper lobe extends to a sharp point and the lower lobe forms a small triangle. Using this description for comparison, other authors erected new species of *Mesacanthus*, but often without providing any new information on the overall anatomy of the genus (Egerton, 1861; Gagnier & Goujet, 1997). Miles (1966) gave *M. pusillus* as the type species for the genus and this was followed by Denison (1979), who also gave a more complete description of the genus overall.

**Figure 1:**
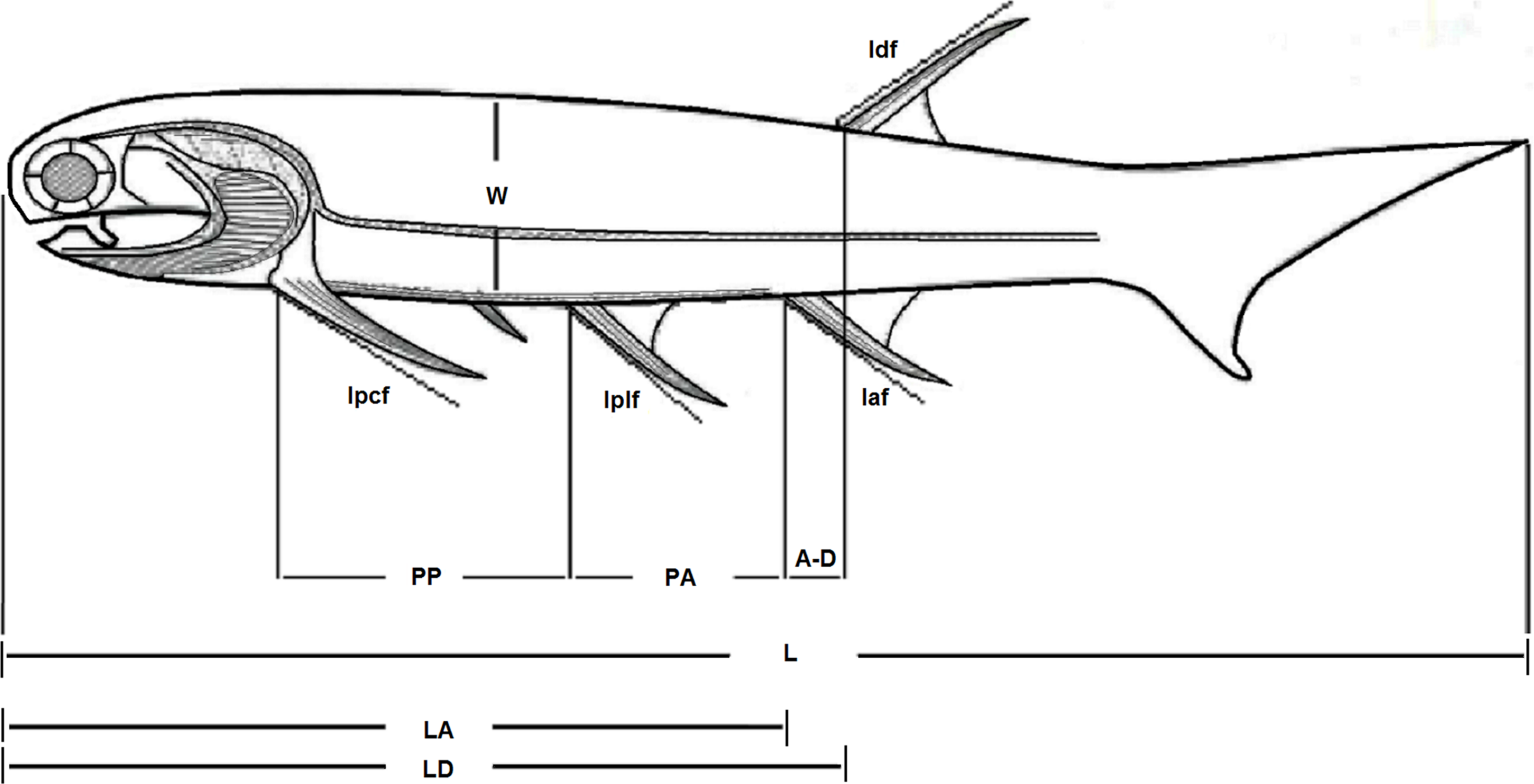
Body plan of the genus *Mesacanthus*. The figure shows the general body plan for all species of the genus *Mesacanthus* and the measurements that were taken and used during this study. Key: L, length; W, width; PP, distance between pectoral to pelvic fin spines; PA, distance between pelvic to anal fin spines; LD, length from front to dorsal fin spine; LA, length from front to anal fin spine; A–D, distance between anal and dorsal fin spines; lPCF, length of the pectoral fin spine; lPLF, length of the pelvic fin spine; lDF, length of the dorsal fin spine; lAF, length of anal fin spine. Adapted from Watson (1937), Fig. 8.

Despite existing for a relatively long amount of time, the genus *Mesacanthus* seems to have changed very little in terms of its overall morphology. Figure 1 shows the general body plan for the genus *Mesacanthus*, which is consistent with the descriptions given by Agassiz (1844–1845), Egerton (1861) and Denison (1979) and can be observed in specimens from both the Lower Old Red Sandstone and the Middle Old Red Sandstone. There have been a number of species proposed for *Mesacanthus* and the most common of these, in both palaeoicthyology collections and in the literature, are *Mesacanthus mitchelli* (Egerton, 1861), *Mesacanthus pusillus* and *Mesacanthus peachi* (Egerton, 1861). These three species were also the only species of *Mesacanthus* recognised by Woodward (1891) after he had formally synonymised *M. peachi* and *M. coriaceus* (Egerton, 1861). In terms of provenance, *M. mitchelli* is found exclusively in the Lower Old Red Sandstone whereas *M. pusillus* and *M. peachi* are found exclusively in the Middle Old Red Sandstone. The oldest of these species stratigraphically, *M. mitchelli*, was described by Egerton (1861) who stated that it was less robust than the Middle Devonian species, *M. peachi*; less robust is interpreted as meaning having a longer, thinner body. A second Middle Devonian species, *M. pusillus*, is also described as being less robust than *M. peachi*, making this species more akin to the Lower Devonian species *M. mitchelli* in this regard. Other than the supposed difference in ‘robustness’ there is little other information in the original descriptions of these species, nor in any of the subsequent literature, that allows for easy differentiation between them in terms of their anatomy. Based upon general morphology alone, it is not possible to easily distinguish between the different species of *Mesacanthus*, especially between those species that are found in the Middle Old Red Sandstone. Whether or not there exist enough anatomical differences between the many known specimens of *Mesacanthus* to justify the assignment of those specimens to three different species is unclear and is the subject of this investigation.

This is the first time that multivariate morphometric analysis has been used to investigate species and genus level taxonomy of acanthodians.

## Material & Methods

The specimens used in this study were taken from a number of collections in the UK including the collections of the University of Aberdeen (ABDUG), the National Museum of Scotland (NMS), the Natural History Museum, London (NHMUK) and the Hunterian Museum (GLAHM). Images of specimens housed in the Elgin Museum (ELGNM), the collections of the British Geological Survey (GMS) and the Royal Ontario Museum, Canada (ROM) were also looked at during the course of this investigation. The specimens include representatives from all three of those species that are most commonly discussed in the literature and cover all of the known localities for *Mesacanthus*. Table 1 shows the number of specimens from each locality, the current specific assignments of those specimens (where specific assignment has been given) and the age (Lower Old Red Sandstone or Middle Old Red Sandstone) of those localities.

**Table 1:** Breakdown of the specimens observed inthis study. The table records the ages, localities, and species identification of the specimens of *Mesacanthus* and *Cheiracanthus* that were observed and measured in this study.

Locality	Group	Species	Number of specimens
Achanarras	Middle Old Red Sandstone	*M. peachi*	34
		*M. pusillus*	6
		*Cheiracanthus sp.*	1
Tynet Burn	Middle Old Red Sandstone	*M. peachi*	1
		*M. pusillus*	9
		*Cheiracanthus sp.*	5
Orkney	Middle Old Red Sandstone	*M. pusillus*	1
Holburn Head	Middle Old Red Sandstone	*M. peachi*	2
Caithness	Middle Old Red Sandstone	*M. peachi*	1
Thurso	Middle Old Red Sandstone	*M. peachi*	2
Cairnfield	Middle Old Red Sandstone	*M. peachi*	1
Tillywhandland	Lower Old Red Sandstone	*M. mitchelli*	21
Duntrune	Lower Old Red Sandstone	*M. mitchelli*	5
Farnell	Lower Old Red Sandstone	*M. mitchelli*	8
Turin hill	Lower Old Red Sandstone	*M. mitchelli*	14
		Total *M. peachi*	41
		Total *M. pusillus*	16
		Total *M. mitchelli*	48
		Total *Cheircanthus sp.*	6
	Total *Mesacanthus*	Middle Old Red Sandstone	**57**
		Lower Old Red Sandstone	**48**
	Total *Cheiracanthus*	Middle Old Red Sandstone	**6**

All of the specimens used in the analyses were studied first hand by the author. A number of key measurements were taken from each of the specimens and these measurements are laid out in Fig. 1, which has been adapted from Watson (1937). Every specimen was also photographed alongside an appropriate scale bar for future reference and comparison. Specimens that were incomplete or showed a large degree of post-mortem deformation, in particular those which had been folded and twisted, but also those in which the borders of the main body were obscured, were discounted from this study on the grounds that the measurements of such things as length and width would not be reliable. In specimens that showed a smaller degree of post-mortem bending, measurements of length and inter-spinal distance were taken along a curved line that was superimposed over the specimen, marked at key points and then straightened. To try and ensure consistency, this curved line was always placed at a distance from the dorsal margin of the body that was equivalent to one third of the width of the specimen at the respective sections along the body (the width of each section was calculated from an average of 3 measurements). This line was placed onto the specimens using a fine thread and the relevant distances were marked on to it at the points of intercept between the thread and lines that ran perpendicular to the dorsal/ventral margins from the locations of the relevant features (e.g. the point of articulation between the pectoral spine and the body). The widths of the specimens (W) that were used in the multivariate analysis and XY scatters were calculated by taking the average of 3 measurements from the middle third of the body. All of the measurements were recorded in a table, along with information on current taxonomic status, locality, preservational status and any other relevant information that could be taken from the specimens and from relevant literature.

The data set was subjected to a series of statistical analyses that included multivariate analysis of variance (MANOVA) canonical variate analysis (CVA) and principle component analysis (PCA), as well as a number of other simple comparisons (Darlington, Weinberg & Walberg, 1973; Jolliffe, 2002). These analyses were carried out using the software PAST (Hammer, Harper & Ryan, 2001) in order to determine whether or not it is possible to separate out the specimens into multiple species using the measurements that were taken.

A simple comparison of the relative lengths and widths of the specimens of each species of *Mesacanthus* was carried out as part of this investigation. This was done because one of the few distinctions between *M. mitchelli*, *M. pusillus* and *M. peachi* that can be found in the current literature is a supposed difference in ‘robustness,’ with *M. peachi* purportedly being ‘more robust’ i.e., having a smaller length to width ratio than *M. pusillus* and *M. mitchelli* (Egerton, 1861). Comparisons between the separation of pectoral, pelvic, anal and dorsal fin spines, as well as the overall shape and absolute lengths of the spines were also carried out to see if any significant differences could be found between the species in terms of those individual measurements as well.

All specimens were considered in this study to belong to the species to which they had been previously assigned. However, a set of tests were also carried out in which previous identifications were disregarded and, following Egerton (1861) and his proposed character of robustness, all specimens were grouped based upon age and length to width (L/W) ratio. This produced three groups: Lower Old Red Sandstone specimens, ‘more robust’ Middle Old Red Sandstone specimens and ‘less robust’ Middle Old Red Sandstone specimens. The Middle Old Red Sandstone specimens of *Mesacanthus* were ordered by length to width ratio and exactly one half were placed into the less robust group and the other half into the ‘more robust’ group. These groups were then subjected to the same MANOVA/CVA and PCA analyses as the rest of the data.

As well as the various analyses in PAST, physical comparisons of the shape and sizes of the pectoral, pelvic, anal and dorsal fin spines of the specimens of *Mesacanthus* were made, as were comparisons of the shape and structure of their scales. Close inspection of these features were carried out using a light microscope and with high resolution photographs to determine if any specific differences could be observed among the specimens in terms of the morphology of these important features.

Data from another acanthodian genus from Scotland, *Cheiracanthus*, was also collected and incorporated into additional analyses in order to test the strength of this method and to test to what degree different groups can be separated using these particular measurements.

## Results

**Figure 2:**
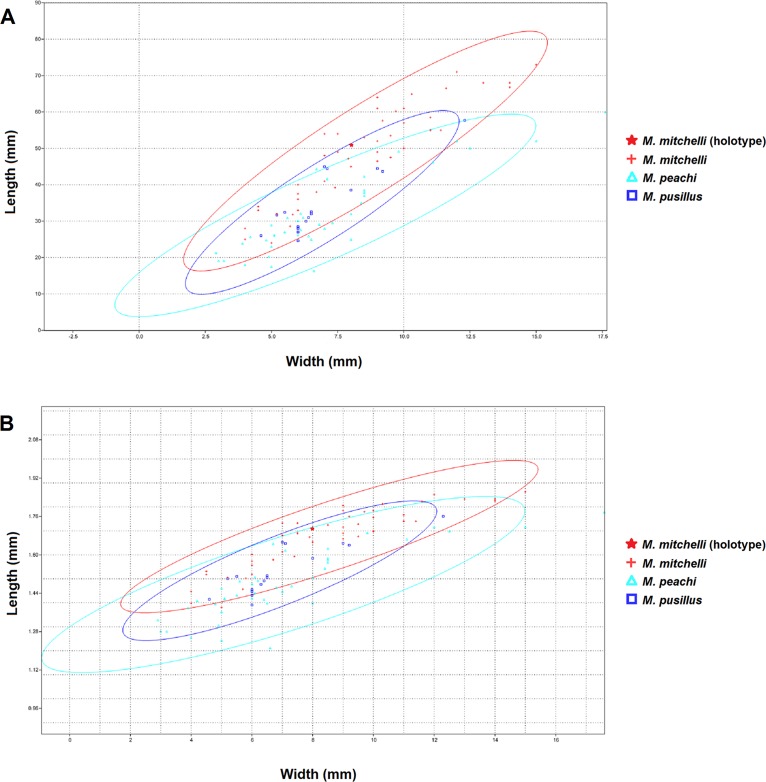
XY scatters of width and length and logtransformed width and length for all 3 species of *Mesacanthus*. The XY scatters for width and length (A) and for log transformed width and length (B) for the three species of *Mesacanthus* show how the specimens from the Lower Devonian (*M. mitchelli*—red crosses) have a slightly higher length to width ratio than the two species from the Middle Devonian (*M. peachi* and *M. pusillus*—light blue triangles and dark blue squares respectively). This demonstrates how the proposed difference in ‘robustness’ between species, as first proposed by Egerton (1861) is at least recovered between specimens from the Lower and Middle Devonian. No difference in robustness is observed between the species from the Middle Devonian.

The bivariate plots produced for length and width (Fig. 2) show that these measurements have a simple linear relationship for all species of *Mesacanthus* (*R*^2^ = 0.7862, 0.7831 and 0.8196 for *M peachi*, *M. pusillus* and *M. mitchelli* respectively). Specimens of *M. mitchelli*, *M. peachi and M. pusillus* do not appear to differ to drastically in these plots, although the 95% confidence ellipses for specimens from the Lower Old Red Sandstone plot marginally higher, meaning that these specimens have on average slightly greater length to width ratios. This difference was investigated further through the application of a *t*-test (Two Sample Assuming Unequal Variance). Table 2 shows the results of t-tests that were carried out on the length to width ratios of the three species, as well as on a number of other anatomical ratios that were calculated from the raw measurements (L/LA, L/LD, (PP + PA)/L—see Fig. 1 for abbreviations). The difference in length to width ratios (L/W) of the specimens of *M. peachi* and *M. pusillus* were found not to be significant (*p*-value = 0.08230). On the other hand, the length to width ratios of the specimens of *M. mitchelli* did show a significant difference to the length to width ratios of the specimens of *M. peachi*, and also of the specimens of *M. pusillus* (*p*-values = 5.55E−08 and 0.000528 respectively: see Table 2). Combining all of the Middle Old Red Sandstone specimens into one category (i.e., treating *M. peachi* and *M pusillus* as synonymous) and comparing their length to width ratios with those of *M. mitchelli* also recovered a significant difference between these two groups (*p*-value = 8.07E−09). It is also worth noting that, of the other combinations of measurements that were subjected to *t*-tests, the sum of the distance between the pectoral and pelvic fin spines divided by the overall length of the specimen ((PP + PA)/L) was found to be significantly different between *M. mitchelli* and *M peachi* (*p*-value = 0.0204) and *M. mitchelli* and *M. pusillus* (*p*-value = 0.0436), but, importantly, not significant between *M. peachi* and *M. pusillus* (*p*-value = 0.755). Again, a significant difference was found between groups of Middle Devonian and Lower Devonian specimens (*p*-value = 8.07E−09). A significant difference was also recovered between the length to anal fin spine and overall length ratio between specimens from the Lower Old Red Sandstone and Middle Old Red Sandstone (*p*-value = 0.0286).

**Table 2:** Results of the *t*-tests (Two Sample Assuming Unequal Variance) that were carried outon the different species of *Mesacanthus* for a number of ratios. The table shows the which ratios were found to show significant differences and non-significant differences between the three species of *Mesacanthus* and between the Middle Devonian specimens and Lower Devonian species when considered as just 2 groups (i.e. when *M. pusillus* and *M. peachi* are considered synonymous). Consistent with the bivariate plots of width and length, the results of these analyses show that a significant difference exists between the L/W ratios of *M. mitchelli* and *M. peachi* and between *M. mitchelli* and *M. pusillus*, but not between *M. peachi* and *M. pusillus*. With the exeption of (PP + PA)/L, no other ratios produced the same distribution of significant and non-significant results among the species and so multivariate analyses using all of the measurements laid out in Fig. 1 were carried out to see if any further differences could be established.

	Group 1	Group 2	|t Stat|	|t Critical two-tail|	Significant difference
**L/W**	*M. peachi*	*M. pusillus*	1.774141	2.015367574	no
	*M. mitchelli*	*M. peachi*	6.028662	1.99167261	yes
	*M. mitchelli*	*M. pusillus*	3.840591	2.034515297	yes
	Lower Devonian	Middle Devonian	6.277312	1.983037526	yes
**(PP** + **PA)/L**	*M. peachi*	*M. pusillus*	0.316376	2.06865761	no
	*M. mitchelli*	*M. peachi*	2.437029	2.034515297	yes
	*M. mitchelli*	*M. pusillus*	2.179869	2.109815578	yes
	Lower Devonian	Middle Devonian	2.821561	2.001717484	yes
**L/LA**	*M. peachi*	*M. pusillus*	0.943376	2.063898562	no
	*M. mitchelli*	*M. peachi*	1.584967	2.008559112	no
	*M. mitchelli*	*M. pusillus*	2.425574	2.109815578	yes
	Lower Devonian	Middle Devonian	2.233208	1.992997126	yes
**L/LD**	*M. peachi*	*M. pusillus*	0.709707	2.051830516	no
	*M. mitchelli*	*M. peachi*	1.83961	2.004879288	no
	*M. mitchelli*	*M. pusillus*	0.920984	2.093024054	no
	Lower Devonian	Middle Devonian	1.867433	1.99167261	no

**Figure 3:**
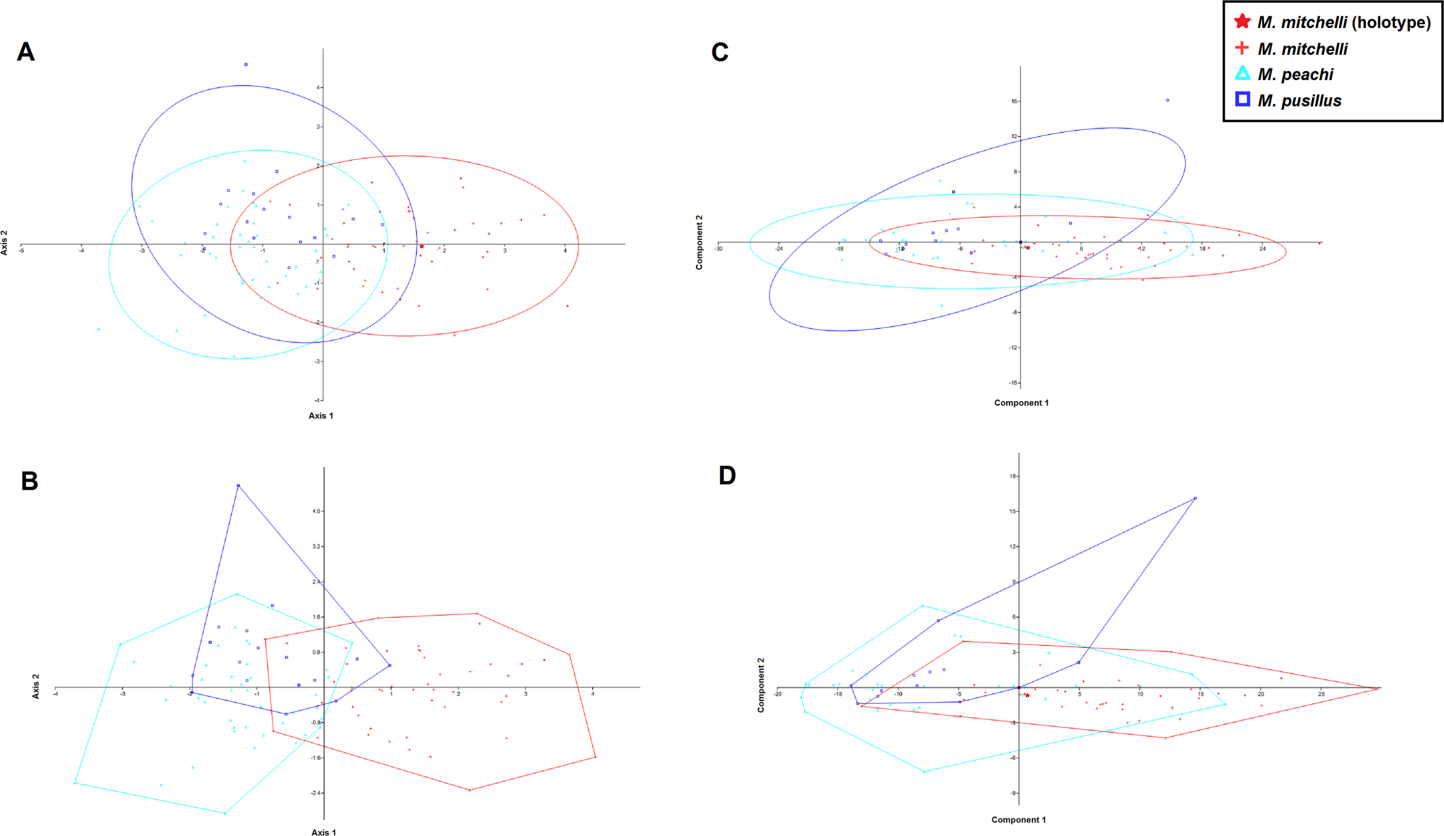
CVA and PCA scatters for all 3 species of *Mesacanthus* with 95% confidence ellipses (A and C) and convex hulls (B and D). CVA scatters (A and B) were produced from the data and show the relative ammounts of overlap between the species of *Mesacanthus*. The Lower Devonian specimens (red crosses) plot predominantly on the positive portion of Axis 2 whereas the the Middle Devonian specimens plot predominantly on the negative postion of Axis 2. The two species from the Middle Devonian (*M. peachi* and *M. pusillus*) show a greater degree of overlap with one another than they do with the Lower Devonian species (*M. mitchelli*). The confusion matrix produced in this analysis is shown in Table 3 along with uncorrected and Bonferonni corrected Hotelling’s *p*-values from pairwise comparions between the groups.

Results of the MANOVA/CVA, in which *M. mitchelli*, *M. pusillus* and *M. peachi* were treated as separate groups, *a priori*, show that *M. peachi* and *M. pusillus* cannot be distinguished from one another based upon the measurements taken, but specimens of *M. mitchelli* can be distinguished from both *M. peachi* and *M. pusillus*. Figures 3A and 3B shows the CVA scatter plot produced and, although there is some overlap between the morphometric profiles of all 3 species, the Lower Devonian species (*M. mitchelli*) does appear to be more separated from the two Middle Devonian species, which overlap with one another to a greater degree; the Middle Devonian specimens plot more along the negative half of Axis 2 whereas the Lower Devonian specimens plot predominantly in the positive half. While this plot may not be completely clear in showing how these groups separate, the numerical results obtained in these analyses show to a much greater degree the amount of overlap and separation between the groups and, crucially, whether or not this type of multivariate analysis recovered any significant differences between the groups based upon the measurements taken. The confusion matrix from this MANOVA test demonstrates the high level of overlap between specimens of *M. peachi* and *M. pusillus* and the relatively low levels of overlap between specimens of these taxa and specimens of *M. mitchelli* (see Table 3). The analyses predicted that 14 of those specimens that are currently identified as *M. peachi* were actually *M. pusillus* and that 7 of those specimens currently identified as *M. pusillus* were *M. peachi* (50% of those analysed in total). This shows a high degree of similarity between specimens of *M. peachi* and *M. pusillus*. Conversely 37 out of 48 of those specimens currently identified as *M. mitchelli* were recovered as such by this analysis (77%). The results of uncorrected and Bonferonni corrected pairwise comparisons between these specimens show that a significant difference exists between specimens of *M. mitchelli* and specimens of both *M. peachi* and *M. pusillus* (Uncorrected *p*-values = 1.03E−12 and 2.24E−05 respectively; Bonferonni corrected *p*-values = 3.10E−12 and 6.73E−05 respectively), but show no that significant difference exists between specimens of *M. peachi* and *M. pusillus* (Uncorrected *p*-values = 0.367; Bonferonni corrected *p*-values = 1: see Table 3).

**Table 3:** Confusion matrix and uncorrected and Bonferonni corrected *p*-values for analyses between 3 species of *Mesacanthus*. The confusion matrix (jackknifed) shows the given groups (rows) and the predicted groups (columns) obtained from the data and highlights the relatively large amount of confusion between specimens of *M. peachi* and *M. pusillus* and the relatively low amount of confusion between those two taxa and *M. mitchelli*. The uncorrected and Bonferonni corrected *p*-values show how significant differences (green) were found between specimens of *M. mitchelli* and *M. pusillus* and between specimens of *M. mitchelli* and *M. peachi*, but no significant difference (red) was found between specimens of *M. peachi* and *M. pusillus*. A significant difference was also found between all specimens from the Lower Devonian and all specimens from the Middle Devonian. These results are consitent with those obtained from the bivariate analyses shown in Table 2.

		*M. peachi*	*M. pusillus*	*M. mitchelli*	**Total**
**Confusion matrix**	***M. peachi***	25	14	2	41
	***M. pusillus***	7	7	3	17
	***M. mitchelli***	4	7	37	48
	**Total**	36	28	42	106
**Uncorrected**	***M. peachi***	–			
	***M. pusillus***	0.367081^b^	–		
	***M. mitchelli***	1.03E−12^a^	2.24E−05^a^	–	
**Bonferonni corrected**	***M. peachi***	–			
	***M. pusillus***	1^b^	–		
	***M. mitchelli***	3.10E−12^a^	6.73E−05^a^	–	

**Notes.**

a Green.

b Red.

The results of the PCA show less of a distinction between the specimens of *Mesacanthus*. Figures 3C and 3D shows PCA plots from analyses that were carried out with *M. mitchelli*, *M. pusillus* and *M. peachi* grouped separately, *a priori*. In this analysis all three species show a great deal of overlap with the other two. Treating Middle Old Red Sandstone and Lower Old Red Sandstone specimens as the only two groups, the PCA again provided only a small amount evidence for a distinction based upon the measurements (see SOM S1). A PCA of just the specimens from the Middle Old Red Sandstone, where *M. peachi* and *M. pusillus* were again grouped separately, also showed a large amount of overlap between the specimens (see SOM S2).

The results of the analyses on the artificially created groups (where Middle Devonian specimens were regrouped based upon ‘robustness’) also showed that, based upon the measurements in this study other than length and width, no significant difference exists between the two groups from the Middle Old Red Sandstone i.e., those that appear ‘more robust’ and ‘less robust’ (Uncorrected *p*-value = 0.994; Bonferonni corrected *p*-value = 1). Importantly, the difference between each of these groups (‘more robust’ and ‘less robust’) and the Lower Old Red Sandstone specimens was found to be significant (Uncorrected *p*-values = 0.000241 and 1.14E−05; Bonferonni corrected *p*-value = 0.000722 and 3.42E−05: see Table 5). This result shows that, regardless of how Middle Old Red Sandstone specimens are grouped, the measurements used in this study do not produce significant differences and therefor do not allow for differentiation between them. In addition to this it shows how Middle and Lower Old Red Sandstone specimens can be differentiated even if current taxonomic identifications are discarded (see Table 5 and Fig. 9).

**Table 4:** Confusion matrix and uncorrected and Bonferonni corrected *p*-values for analyses between specimens of *Mesacanthus* and *Cheiracanthus*. The table shows the confusion matrix produced in the analyses that also included Cheiracanthus. As in Table 3, there is a relatively high ammount of confusion between specimens of *M. peachi* and *M. pusillus*. The uncorrected and Bonferonni corrected Hotelling’s *p*-values show how there are significant differences (green) between all the species except for between *M. peachi* and *M. pusillus*, which are non-significant (red).

		*M. peachi*	*M. pusillus*	*M. mitchelli*	*Cheiracanthus sp.*	**Total**
**Confusion matrix**	***M. peachi***	19	15	7	0	41
	***M. pusillus***	9	5	3	0	17
	***M. mitchelli***	7	4	37	0	48
	***Cheiracanthus sp.***	0	2	0	4	6
	**Total**	35	26	47	4	112
**Uncorrected**	***M. peachi***	–				
	***M. pusillus***	0.657332^b^	–			
	***M. mitchelli***	1.83E−07^a^	0.00462002^a^	–		
	***Cheiracanthus sp.***	6.93E−15^a^	1.79E−05^a^	1.01E−17^a^	–	
**Bonferonni corrected**	***M. peachi***	–				
	***M. pusillus***	1^b^	–			
	***M. mitchelli***	1.10E−06^a^	0.0277201^a^	–		
	***Cheiracanthus sp.***	4.16E−14^a^	0.000107343^a^	6.05E−17^a^	–	

**Notes.**

a Green.

b Red.

**Table 5:** Confusion matrix and uncorrected and Bonferonni corrected *p*-values for analyses between artifical groups of *Mesacanthus*. The table shows the confusion matrix produced in the same analysis that produced Fig. 9, in which the specimens of mesacanthus were sorted into groups based upon age and ‘robustness’, disregarding previous specific identifications. Group 1, ‘less robust’ Middle Old Red Sandstone specimens; Group 2, ‘more robust’ Middle Old Red Sandstone specimens; Group 3, Lower Old Red Sandstone specimens (=*M. mitchelli*). Red, not significant; Green, significant.

		Group 1	Group 2	Group 3	Total
**Confusion matrix**	**Group 1**	9	16	4	29
	**Group 2**	20	7	2	29
	**Group 3**	15	4	29	48
	**Total**	44	27	35	106
**Uncorrected**	**Group 1**	–			
	**Group 2**	0.99374^b^	–		
	**Group 3**	0.000241^a^	1.14E−05^a^	–	
**Bonferonni corrected**	**Group 1**	–			
	**Group 2**	1^b^	–		
	**Group 3**	0.000722^a^	3.42E−05^a^	–	

**Notes.**

a Green.

b Red.

Comparison of the morphologies of the various fin spines of *Mesacanthus* showed that some difference did exist between certain specimens in terms of the curvature of these features; some specimens had curved fin spines while other had very straight fin spines. However, further investigation of this potential character found that fin spine curvature was actually randomly distributed throughout the various species and no pattern could be seen between fin spine curvature and specific identification. Further to this, investigation of the closely related acanthodian genus *Cheircanthus* also revealed how fin spine curvature was something that did not directly correlate with any of the data gathered as part of this study (size, specific identity, locality etc.). This suggests that these differences in fin spine curvature are not specific differences, but are more likely to be a result of intraspecific variation or even preservation.

Close examination of the scales of *Mesacanthus* under a light microscope and using high resolution photographs also showed that there were no differences between specimens of different species. The scales in all specimens had a similar morphology; small, tightly packed and unornamented and diamond shaped (contra Agassiz, 1844–1845: see Fig. 7). This morphology was homologous along the length of the body, with only small changes in size being observed in the scales of anterior end. Figure 7 shows this scale morphology and arrangement as observed in numerous specimens, including representatives of all three species of *Mesacanthus* looked at in this study. Given the lack of ornamentation, or any other small morphologies, as has been observed in the scales and spines of the specimens of *Mesacanthu*s, as well as a lack of variation in shape along the length of the body, this study did not take the study of the scale and spine micro-structure to any greater degree of sophistication than what is described. Because the scales all appear to lack ornamentation it seems unlikely that any further information would be gained from using greater degrees of magnification that would be of relevance to this particular investigation. Future work on acanthodian scales using SEM imaging may reveal previously undescribed micro-structures in specimens of *Mesacanthus* not visible using only a light microscope and these may prove to be useful in subsequent taxonomic discussions if clear groups can be created on the presence/absence of these micro-structures. However, given there is no mention of differences in scale and spine morphology in the original diagnosis of any of the species other than *M. pusillus*, or in any of the subsequent literature, and that the overall spine and scale morphology has been observed not to differ among any of the specimens looked at in the study, this investigation finds that no morphological differences exist between the scales and spines of *M. mitchelli*, *M. peachi* and *M. pusillus*.

**Figure 4:**
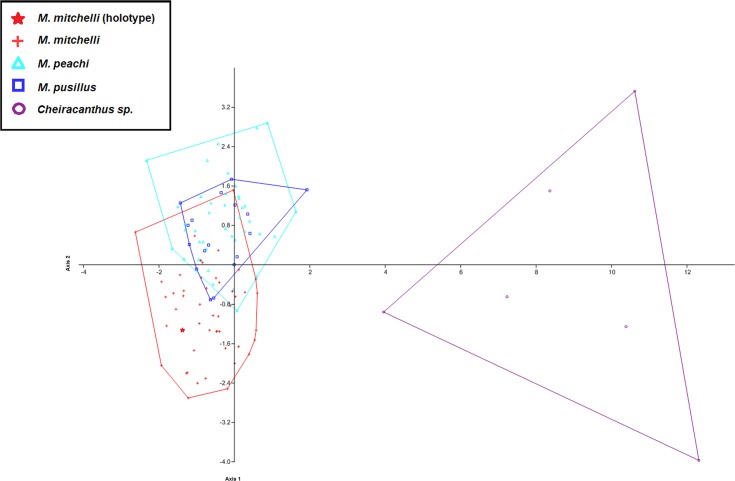
CVA scatter for specimens of *Mesacanthus* and *Cheiracanthus* with convex hulls. The figure shows the high degree of separation between specimens of Cheiracanthus and *Mesacanthus* that is produced using this method with the measurements laid out in Fig. 1. Given how anatomically similar these two genera are, this analysis lends evidence to the strength of this method in helping to distinguish between similar and relatively conservative taxa such as acanthodians. As in Fig. 3, the speciemens of *Mesacanthus* from the Middle Devonian plot more closely to one another than they do to the speicmens from the Lower Devonian. The numerical results of these analyses are shown in Table 4.

**Figure 5:**
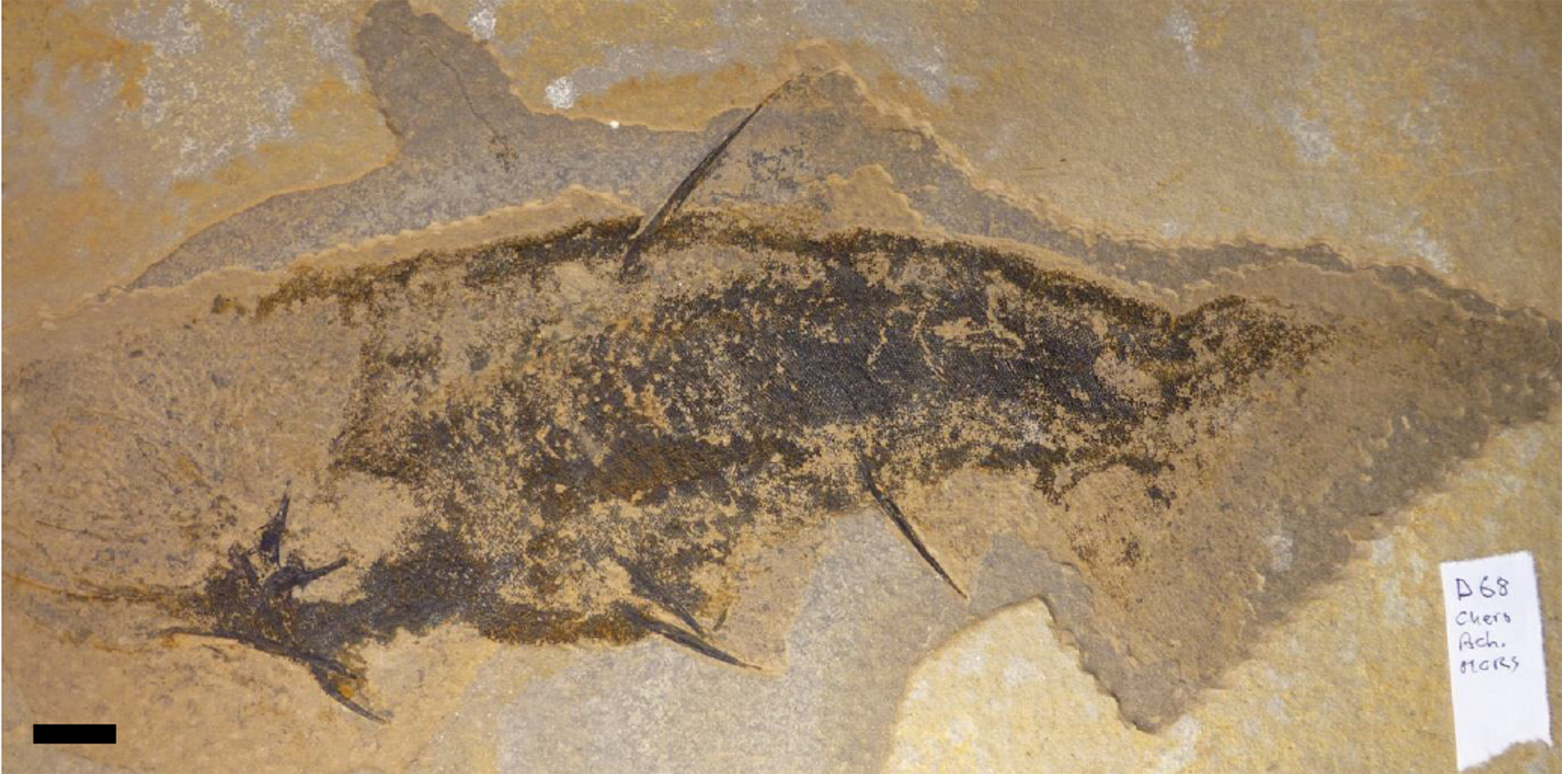
Specimen of Cheiracanthus sp. Specimen ABDUG Pal.D68, from the Middle Old Red Sandstone at Achanarras quarry.

**Figure 6:**
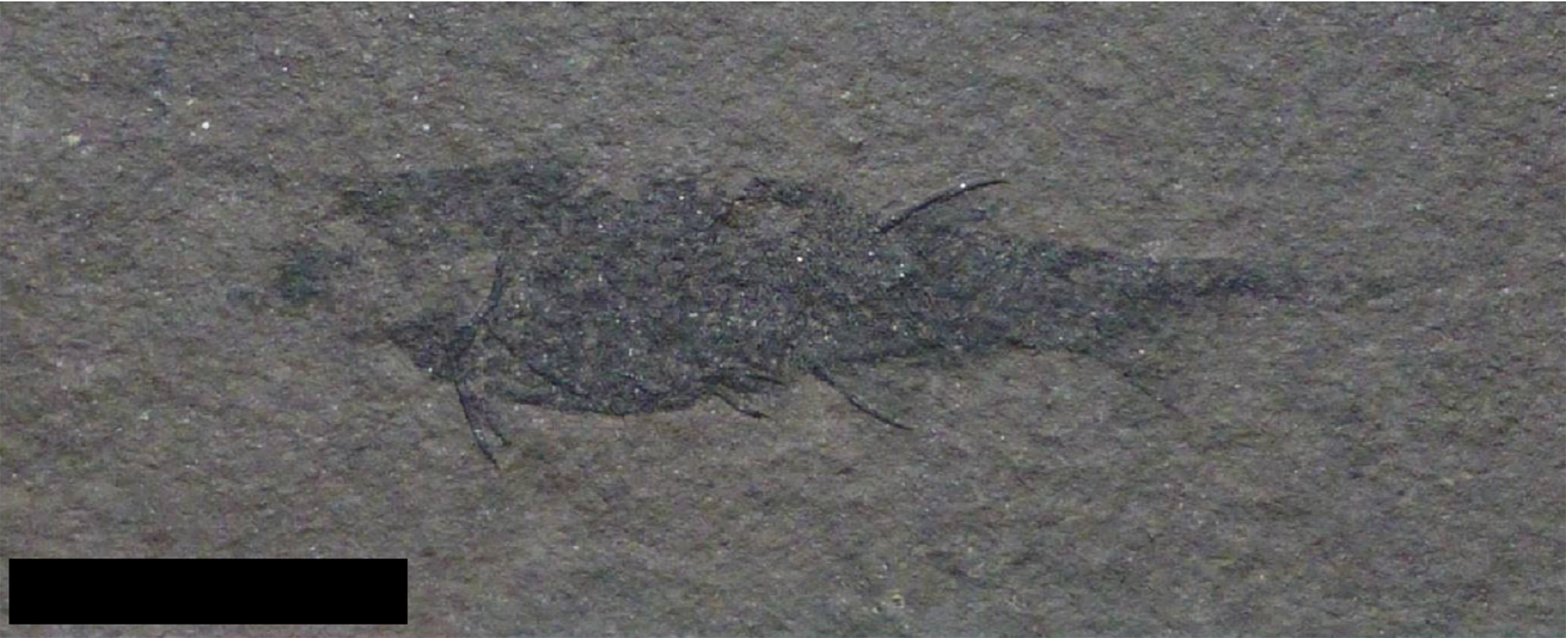
Specimen previously identified as *Mesacanthuspusillus*. Specimen ABDUG Pal.D90 from the Middle Old Red Sandstone of Achanarras quarry showing th e standard body plan for *Mesacanthus* as outlined in Fig. 1. This body plan is the same in all specimens of *Mesacanthus* with no observed differences between the 3 species looked at in this study. Scale bar = 20 mm.

**Figure 7:**
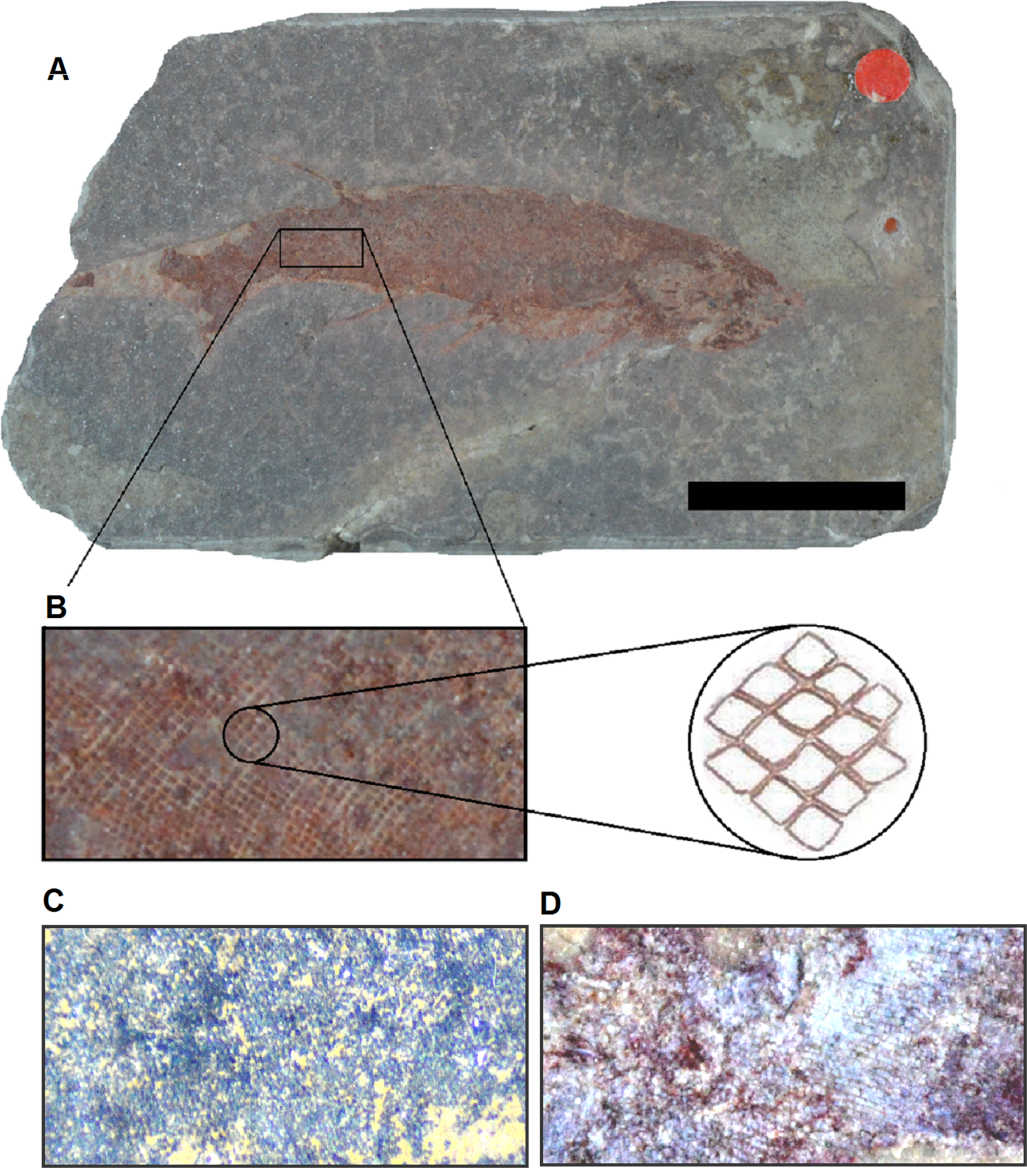
Scale morphology and arrangement in *Mesacanthus*. (A) Specimen NHMUK PV P1331 from the Lower Old Red Sandstone of Turin hill. This specimen is identified as *Mesacanthus mitchelli*. (B) Close up of scales from the posterior third of NHMUK PV P1331. When looked observed under a light microscope, these scales appear to be diamond shaped rather than triangular, and this is true of the scales at every point along the length of the body. Overall the morphology and arrangement is relatively homogenous around the different regions of the body. This same arrangement and scale morphology has been observed in specimens of all 3 species looked at in this study. None of the specimens showed ornamentation on the scales of the main body, head or tail and no other shapes or arrangments could be found that might help distinguish between the species. (C) Close up of scales from specimen NHMUK PV P61697 (*M. peachi*). These scales are unornamented and have been observed to share the same diamond shape and tight packing as the scales in NHMUk PV P1311. (D) Close up of the scales of NHMUK PV P35786 (*M. pusillus*).

To investigate whether or not this method can be effective in differentiating between morphologically similar taxa a second set of MANOVA/CVA tests were carried out that also incorporated data from the genus *Cheiracanthus*, another acanthodian from the Devonian of Scotland (see Fig. 5). This analysis showed how clearly specimens of *Cheiracanthus* can be distinguished from specimens of *Mesacanthus* using the same set of measurements (see Fig. 4). Table 4 shows the confusion matrix produced in this analysis of *Cheiracanthus* and *Mesacanthus* as well as the Uncorrected and Bonferonni corrected Hotelling’s *p*-values that were recovered. Given the similarity between these two genera these results highlight just how effective the measurements used in this study can be in discriminating between different taxonomic groups. Further to this, a PCA was carried out in which specimens of the genus *Cheiracanthus* were also included. Just as with the CVA results, the PCA results show a great deal of separation between the specimens of *Cheiracanthus* and the specimens of *Mesacanthus* (see SOM S3) lending further evidence to the high potential of this method as a tool for discriminating between similar, anatomically conservative taxa.

**Figure 8:**
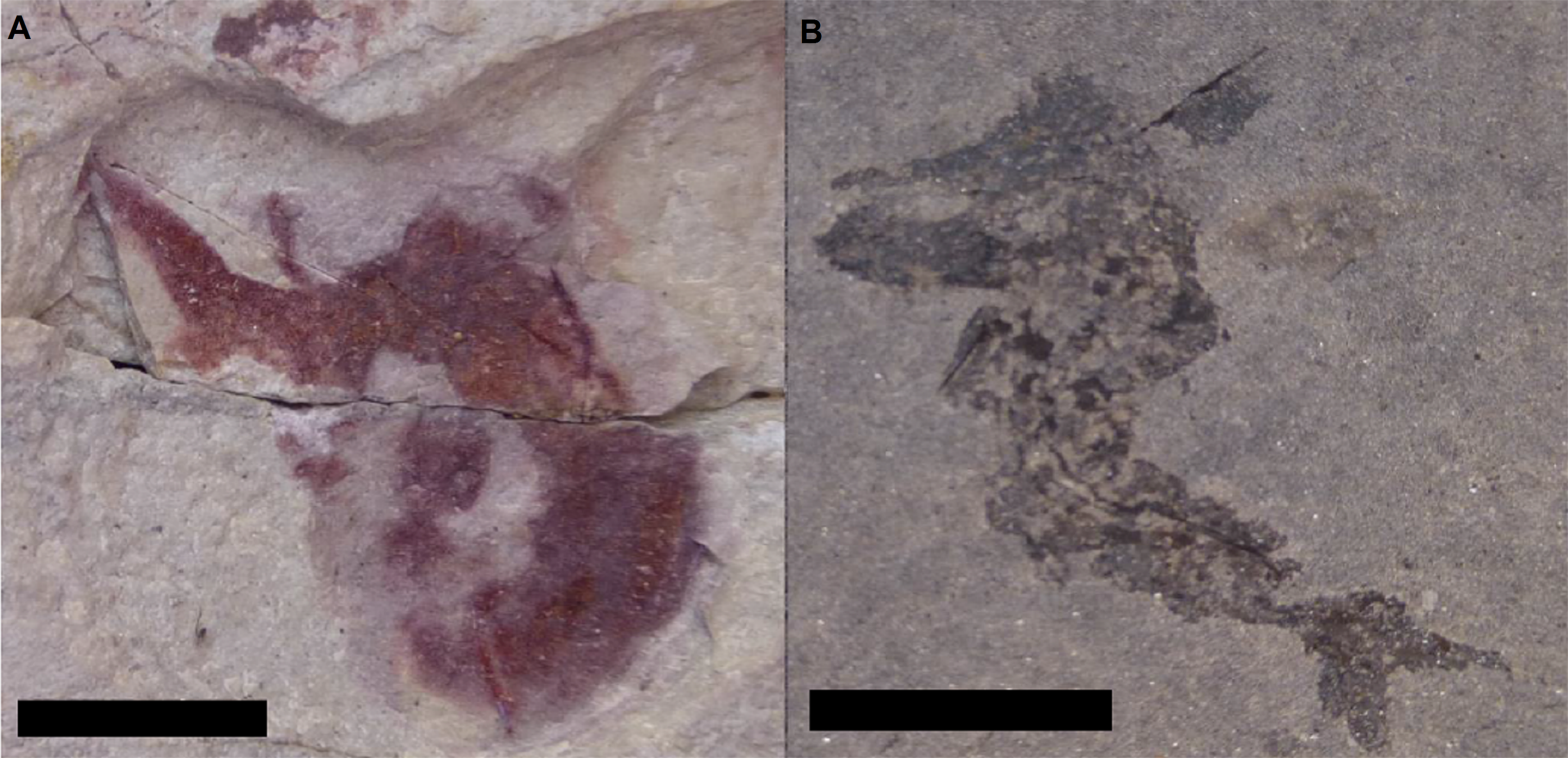
Preservational differences between localities that have produced specimens of *Mesacanthus peachi*. Specimens of *M. peachi* from Tynet Burn (A) and Achanarras (B) showing the differences in preservation between the areas. Both specimens show a high degree of post mortem damage (folding and twisting) and so neither was suitable for use in this study. Scale bar = 20 mm.

In all of the analyses, the holotype of *M. mitchelli* (NHMUK PV P560) plotted well within the standard range for the species; it did not plot close to specimens of *M. peachi* and *M. pusillus* (see Figs. 2, 3A–3D, 4 and 8).

## Discussion

Multivariate morphometric analyses have been widely used in the fields of botany, zoology and palaeontology for the purposes of taxonomic investigation and species discrimination (LaFlamme, Narbonne & Anderson, 2004; Henderson, 2006; Wang et al., 2011; Mayo & Andrade, 2013; Zylinska, Kin & Nowicki, 2013; Jagersbacher-Baumann, 2014). This study utilised PCA and MANOVA/CVA to investigate the amount variation among multiple species within a genus, which has previously been done for genera from a very broad range of organisms, from flowering plants (Mayo & Andrade, 2013) to mites (Baker & Schwarz, 1997; Jagersbacher-Baumann, 2014; Navia et al., 2015). This method has also been used in numerous taxonomic investigations into genera of extant fish (e.g. Maderbacher et al., 2008; Cooper et al., 2014).

The strength of these methods in discriminating between anatomically similar acanthodians, using the measurements outlined in Fig. 1, was tested through the addition of the genus *Cheiracanthus* into the data set. *Mesacanthus* and *Cheiracanthus* differ in a number of morphological ways (scale morphology, presence/absence of prepelvics) but in terms of the measurements used in this study the only observed differences are in the total body length and width (specimens of *Mesacanthus* are on average approximately 35% the average length of specimens of *Cheiracanthus*and only 25% the average width) and the relative positions of the attachments for the dorsal and anal fin spines. In terms of the latter character, specimens of *Cheiracanthus* differ in the fact that the attachment of their dorsal fin spine is located anterior to the attachment of their anal fin spine, and not the other way around, as is the case in all species of *Mesacanthus* (i.e., LD:LA for *Cheiracanthus* <1.0 and LD:LA for *Mesacanthus* >1.0). Given how similar these genera are overall, the results of the various bivariate and multivariate analyses that included specimens of *Cheiracanthus* showed a clear distinction between the two taxa. Because of the results of these additional analyses the author feels that this method does have potential when it comes to being used for discrimination between similar and morphologically conservative taxa such as acanthodians.

Given that *M. mitchelli* is currently said to be distinguishable from *M. peachi* on the basis of how ‘robust’ the two species are (*M. peachi* being supposedly more ‘robust’ than *M. mitchelli*) then a difference in the XY scatters of length and width between specimens of *M. mitchelli* and *M. peachi* should be expected. Simple comparison of these features shows that such a difference does exist and that it is significant (see Table 2). Given that *M. peachi* is also described as being more robust than *M. pusillus*, we would expect specimens of *M. pusillus* to plot separately from the specimens of *M. peachi*, somewhere closer to specimens of *M. mitchelli*, on the same XY scatter. However, we do not see this in Fig. 2. In addition to this, no significant difference in the lengths and widths between specimens of these two Middle Old Red Sandstone species was found (see Table 2). From this we must conclude that, among the specimens from the Middle Old Red Sandstone, there is no significant difference in the length to width ratios, and therefore no difference in ‘robustness.’ Given that a difference in ‘robustness’ is currently the only character described that can be used to separate specimens of *Mesacanthus* from the Middle Old Red Sandstone into two species, *M. pusillus* and *M. peachi*, this result strongly suggests that the specimens of *Mesacanthus* from the Middle Old Red Sandstone probably represent only a single species and not two distinct species, as has previously been thought. Combining this result with the results of the MANOVA/CVA and PCA, it seems apparent that there is not enough evidence to justify the separation of Middle Old Red Sandstone specimens into two distinct species based purely upon the overall shape and size of the specimens.

The MANOVA/CVA that were carried out in which the Middle Devonian specimens were grouped based solely on L/W and not by previous taxonomic assignments also produced no significant differences between the two groups (i.e., the ‘more robust’ group and the ‘less robust’ could not be discriminated based upon any of the other measurements: see Table 5). Importantly, both groups still showed a significant difference with *M. mitchelli*. This demonstrates how, regardless of how the Middle Devonian specimens are grouped, the Lower and Middle Devonian specimens are significantly different from one another based upon the measurements taken and used in these multivariate analyses.

Additionally, multivariate analyses which grouped all of the Middle Devonian specimens into a single group (i.e., treated *M. peachi* and *M. pusillus* as synonymous) found a significant differences between this group and the specimens of *M. mitchelli* (see SOM S1, S4 and S5). This Middle Devonian group also differed significantly from specimens of *Cheiracanthus* (Uncorrected *p*-value = 7.96E−20; Bonferonni corrected *p*-value = 2.65E−20).

The bivariate and multivariate analysis were carried out following a more general study of the anatomical features of the specimens that included investigation of the morphology of the scales and fin spines. These anatomical investigations yielded no clear distinguishing characters as all three species of *Mesacanthus* were found to share the same scale and spine morphology. The scales were found to be tightly packed and diamond shaped along the full length of the body with only subtle change in dimensions occurring between various sections. Investigations with a light microscope revealed no ornamentation was present on the scales of any specimens of *Mesacanthus* and thus this type of acanthodian character cannot be used here to make any further discrimination between specimens. However, in the future, more sophisticated techniques such as SEM imaging may yield new information the scale and spine structure that may help better split or group together different taxa within Acanthodii, including *Mesacanthus* species.

However, as this study found no anatomical differences between specimens other than those which were recovered from the bivariate analyses of bodily dimensions, the results of the multivariate analysis must also be taken into consideration when discussing the validity of the taxa looked at in this study. Given that no evidence was found in any of the studies (morphological and morphometric) for the existence of two distinct Middle Devonian groups within the specimens, the current taxonomic distinctions within *Mesacanthus* should be reviewed.

Seeing as the name *M. pusillus* was in use first (Agassiz, 1844–1845) it must take priority over *M. peachi*. Egerton (1861) gave the only description which tried to diagnose and differentiate between the two species from the Middle Old Red Sandstone, and this was primarily focused around a perceived level of ‘robustness’. Given that this is currently the only proposed discriminating character, and that no evidence has been found to support such a distinction, the species *M. peachi* must be provisionally regarded as invalid, on the grounds that the taxa was erected after *M. pusillus*. This makes *M. peachi* a junior subjective synonym of *M. pusillus*. On the other hand, evidence was found for the Lower Old Red Sandstone species, *M. mitchelli*, being less robust than specimens from the Middle Old Red Sandstone. The subsequent multivariate analyses also recovered a significant difference between the specimens from the Lower Old Red Sandstone and the Middle Old Red Sandstone. This partially supports the claim made by Egerton (1861) that some Middle Old Red Sandstone specimens (those that he called *M. peachi*) were ‘more robust’ than *M. mitchelli*. Despite the fact that Egerton (1861) was describing *M. peachi* and not *M. pusillus* when he gave that diagnosis, and also went on the try and discriminate between the two Middle Old Red Sandstone taxa using the same character, the fact that the chosen discriminating character has been shown to not be sufficient and that no other differences could be found between the specimens from the Middle Old Red Sandstone either, it must be concluded that all specimens from the Middle Old Red Sandstone should be regarded as belonging to one species, *M. pusillus*. From this evidence, it seems reasonable to retain the names *M. mitchelli* and *M. pusillus* for specimens from the Lower Old Red Sandstone and Middle Old Red Sandstone respectively. Further investigation of these taxa may yield more differences in their anatomy but provisionally this study diagnosis them using the same character as Egerton (1861), that is, *M. mitchelli* has a greater length to width ratio than *M. pusillus*.

The holotype of *M. mitchelli* (NHMUK PV P560) was studied first hand by the author and data taken from it was used in the analyses of this investigation. The current whereabouts of the holotype specimens of *M. peachi* and *M. pusillus*, however, remain uncertain, despite an exhaustive search of the literature and collections. Andrews (1982) regarded the syntypes of *M. pusillus* lost but also went on to state that certain specimens in the collections of the Royal Ontario Museum and in the Elgin Museum could possibly be the counterparts to the syntype material. This investigation and has identified two specimens in the collection of the Royal Ontario Museum and one specimens in the Elgin Museum that could be the missing syntype material (or the counterpart of it) of *M. pusillus*. Agassiz (1844–1845, pl. 28, Figs. 8–10) figured three specimens when describing *Acanthodes pusillus* and two of these figured specimens, although the figures are not very clear, closely resemble specimens ROM 25872, ROM 25846 and ELGNM 1978.191.1 (see SOM S7). It could be possible that ELGNM 1978.191.1 represents the counterpart to the holotype, as was also proposed by Andrews (1982). However, due to a lack of clarity on which specimen is which, as well as the high level of deformation in the specimens, these were not used in the analyses carried out in this study. The holotype of *M. peachi* is also not currently accounted for Egerton (1861) provided a figure with his original description of the species (although he did not state explicitly that the figured specimen is the holotype) and this figure has been used to try and identify the holotype specimen in this study (Egerton, 1861, pl. 6, Fig. 1). The figure SOM S6 shows specimen GSM 21448 from the collections of the British Geological Survey, which appears to be the specimen that Egerton (1861) figured in his original description. However, Egerton (1861) did not state explicitly that the specimen figured in the original description was the holotype and so it is impossible to say from the current information available whether or not GSM 21448 is the holotype or not. In the future it may prove to be necessary to designate a neotype from the current material. However, this study will not designate a neotype at this time in order to avoid future taxonomic confusion should the holotype ever remerge.

**Figure 9:**
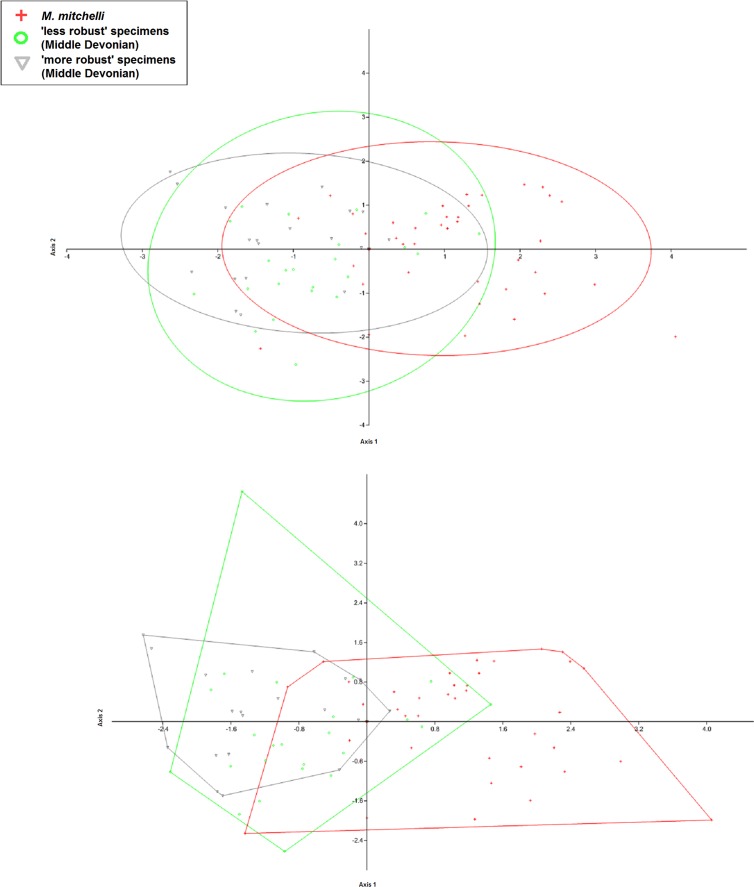
CVA scatter produced when previous specific identifications are disregarded and the specimens regrouped based upon L/W ratio and age only. The figure shows the CVA scatter produced when the specimens were grouped using age and ‘robustness’ only. This was done in order to determine if the chracter of ‘robustness’ as first described by Egerton (1861) could be used to group the specimens, especially those from the Middle Devonian, that then could be shown to differ significant from one another in terms of the other measurments taken in this study using multivariate analyses. As in Figs. 3A and 3B, there is a greater amount of overlap between the 2 different groups from the Middle Devonian than there is between those groups and the group from the Lower Devonian, with Middle Devonian specimens again plotting in the negative portion of Axis 2 and the Lower Devonian specimens plotting predominantly in the positive portion of Axis 2. As only 1 species is reported fom the Lower Devonian, these specimens are still listed here as *M. mitchelli*. The fact that even Middle Devonian groups that are artificially created using the only specifically differentiating character currently given in the literature do not appear differ significantly from another shows that there is not enough evidence for 2 distinct species of Mesacanthus in the Middle Old Red Sandstone. The confusion matrix produced in this analysis as well as the uncorrected and Bonferonni corrected Hotelling’s *p*-values are shown in Table 5.

## Conclusions

We conclude that the genus *Mesacanthus* contains 2 valid species from in the Orcadian Basin and Midland Valley areas of Scotland and that those species are *M. mitchelli* and *M. pusillus*. The two distinct species come from different times in the region’s history, one from the Lower Devonian and one from the Middle Devonian. This study has found that *Mesacanthus mitchelli*, from the Lower Devonian, has a lower width to length ratio (is less robust) than *Mesacanthus pusillus*, from the Middle Devonian. This study has also concludes that *M. pusillus* should provisionally be considered the only valid species name for specimens from the Middle Devonian, as no evidence was found for a second, distinct Middle Devonian species, *M. peachi*, as was originally stated by Egerton (1861).

## Supplemental Information

10.7717/peerj.1331/supp-1 SOM S1 PCA scatters for specimens from the Lower Old Red Sandstone and Middle Old Red Sandstone with convex hulls (above) and 95% confidence ellipses (below) The figure shows the large amount of ovelap between specimens from the Middle Devonian (pink squares) and Lower Devonian (grey inverted triangles) recovered by the PCA. Specimens from the Middle Devonian occupy a greater range of positions within the morphospace but oveall do not differ drastically from the specimens from the Lower Devonian in this analysis.

10.7717/peerj.1331/supp-2 SOM S2 PCA scatter for Middle Old Red Sandstone species of *Mesacanthus* with convex hulls The PCA scatter shows the morphospace occupied by the 2 Middle Devonian species *M. peachi* and *M. pusillus*. As in Fig. 6, there is a large amount of overlap beween the 2 groups in this particular analysis with all but one specimen (NHMUK PV P3578b) of *M. pusillus* falling within the same space as the specimens of *M. peachi*.

10.7717/peerj.1331/supp-3 SOM S3 PCA scatter for *Mesacanthus* and Cheiracanthus As in Fig. 4, this figure shows how this analysis produced a great degree of sepeartionbetween specimens of *Mesacanthus* and Cheiracanthus. Again, this lends evidence to thestrength of this method and its potential for use in future studies of other similar taxa.

10.7717/peerj.1331/supp-4 SOM S4 CVA scatter for Cheiracanthus and Middle Old Red Sandstone and Lower Old Red Sandstone specimens of *Mesacanthus* with convex hulls The figure shows the CVA scatter that is produced when the specimens of *Mesacanthus* are divided into 2 groups (Middle Devonian specimens and Lower Devonian specimens) and analysed alongside specimens ofCheiracanthus. The confusion matrix produced in this analysis and the uncorrected and Bonferonni corrected Hotelling’s *p*-values are shown in Table 6.

10.7717/peerj.1331/supp-5 SOM S5 Confusion matrix and uncorrected and Bonferonni corrected *p*-values for analyses between specimens of Cheiracanthus and Middle Devonian and Lower Devonian *Mesacanthus* specimens The confusion matrix shows the relatively low amount of confusion between specimens from the 3 groups in this particular analysis. The uncorrected and Bonferroni corrected Hotelling’s *p*-values show how each of these groups was found to be significantly different from the others. This is important because this shows that when *M. peachi* and *M. pusillus* are synonymised the single taxon is found to still be significantly different from other acanthodian taxa. Red = not significant; Green = significant.

10.7717/peerj.1331/supp-6 SOM S6 Possible holotype specimen for *Mesacanthus* peachi (A) Figure from Egerton (1861, pl. 6 Fig. 1) which is the only figure given with the original description of *M. peachi*. (B) Specimen GSM 21448, which is very likely the specimen figured by Egerton (1861) despite the disparity in the size of the slab it appears on and of the slab depicted in the illustration. Scale bar = 20 mm.

10.7717/peerj.1331/supp-7 SOM S7 Possible holotype material for *Mesacanthus pusillus* (A) One of three figures given by Agassiz (1844–1845, pl. 28, Figs. 8–10) with the original description of *M. pusillus*. (B) Specimen ROM 25846, which is possibly one of the specimens (or counterpart to) that was figured by Agassiz (1844–1845). (C) Another of the three figuresgiven by Agassiz (1844–1845). (D) Specimen ELGNM 1978.191.1, which could also be one of the specimens (or counter part to) that was figured by Agassiz (1844–1845). (E) Specimen ROM 25872, which could also be one of the specimens (or counter part to) that was figured by Agassiz (1844–1845). Scale bar = 20mm.

10.7717/peerj.1331/supp-8 SOM S8 PCA Eigenvalue and % variance for the various analyses

10.7717/peerj.1331/supp-9 Supplemental Information 1 Raw Data The table records all of the measurements taken for each specimen looked at in this study.
