# Supplementary figures and images for: An investigation of the genus Mesacanthus (Chordata: Acanthodii) from the Orcadian Basin and Midland Valley areas of Northern and Central Scotland using traditional morphometrics
Source: PeerJ. 2015 Oct 29;3:e1331. doi: 10.7717/peerj.1331 (PMC4631467; doi:10.7717/peerj.1331)

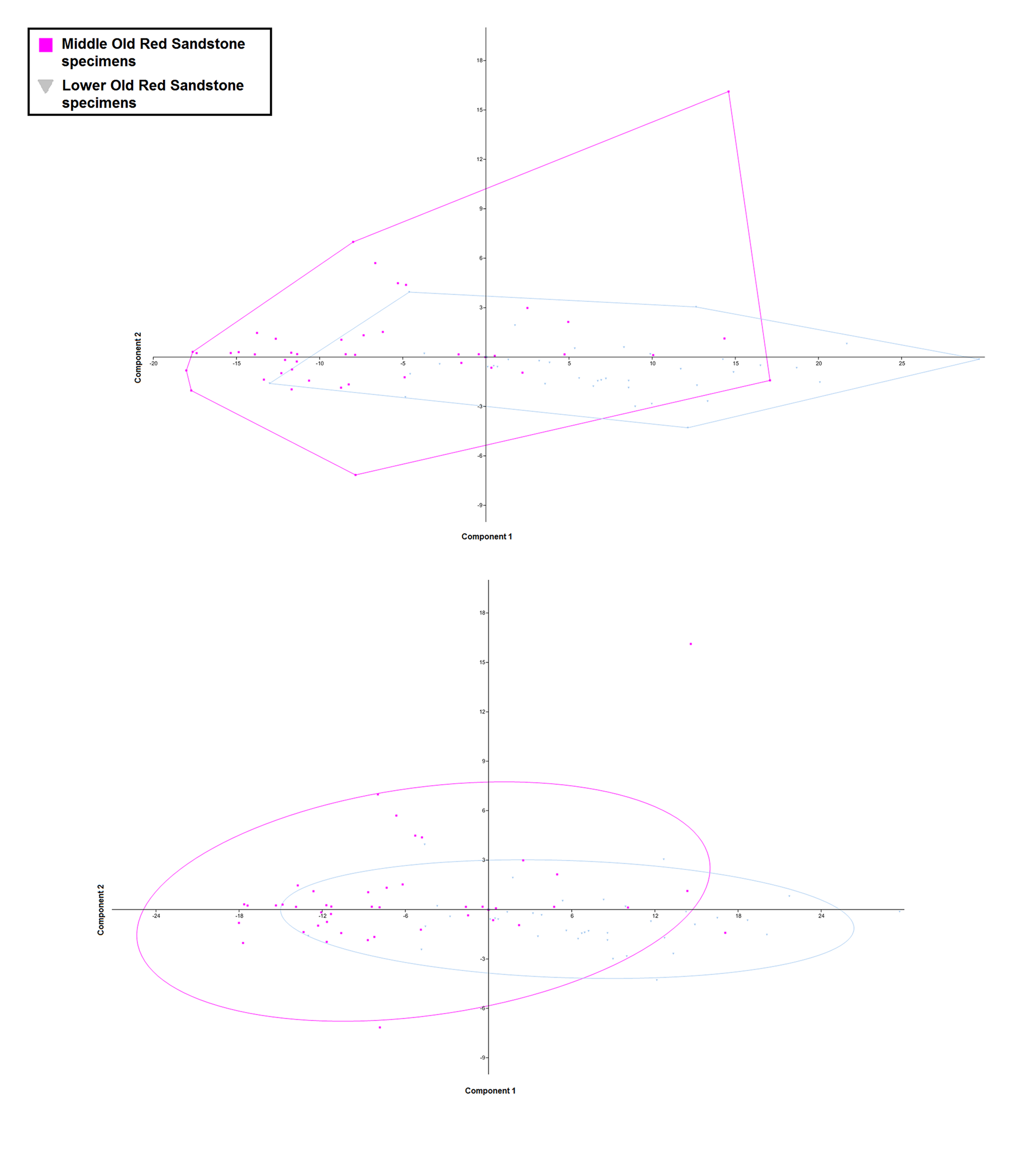

Supplement: SOM S1 — The figure shows the large amount of ovelap between specimens from the Middle Devonian (pink squares) and Lower Devonian (grey inverted triangles) recovered by the PCA. Specimens from the Middle Devonian occupy a greater range of positions within the morphospace but oveall do not differ drastically from the specimens from the Lower Devonian in this analysis. [file peerj-03-1331-s001.png]

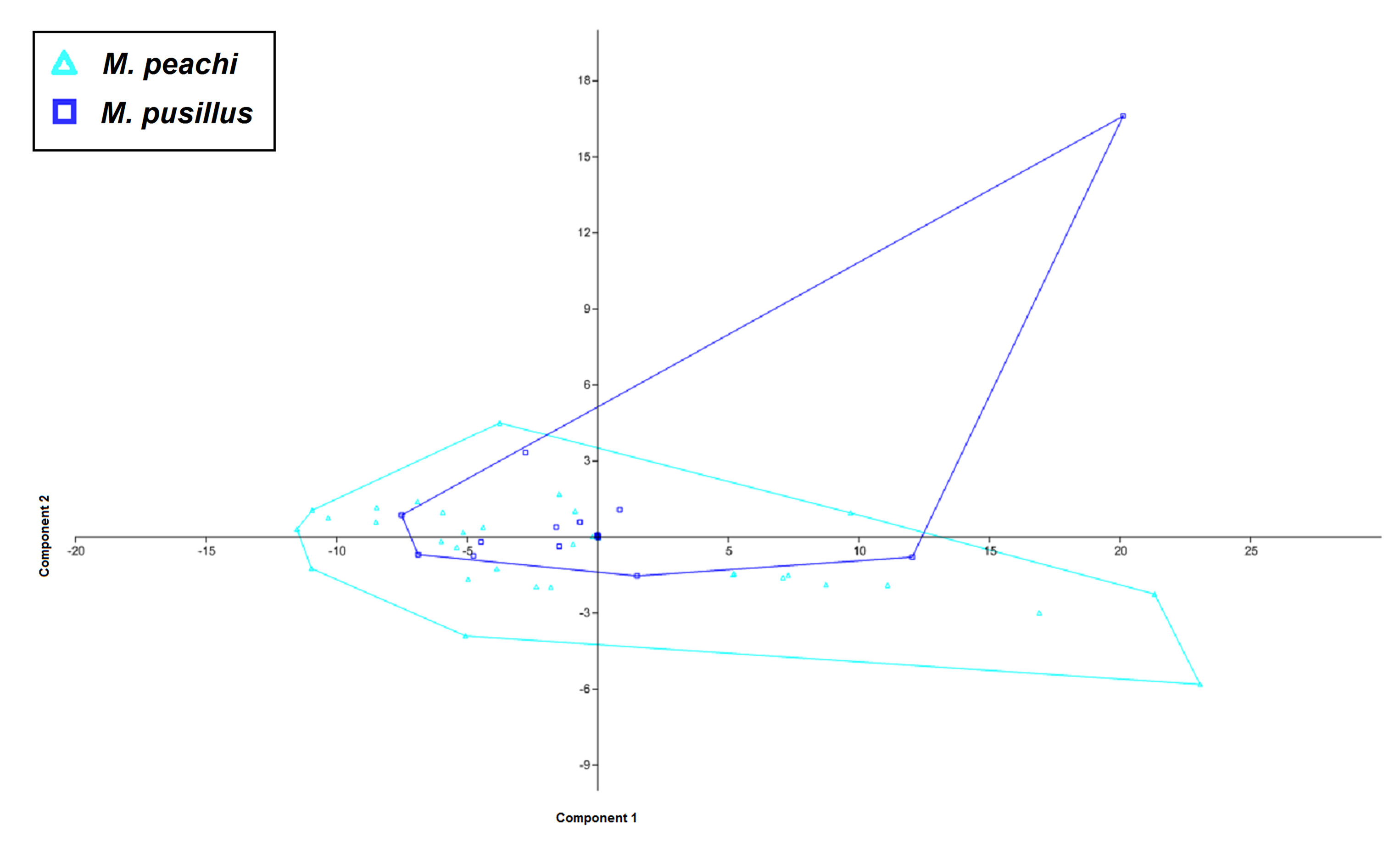

Supplement: SOM S2 — The PCA scatter shows the morphospace occupied by the 2 Middle Devonian species M. peachi and M. pusillus. As in Fig. 6, there is a large amount of overlap beween the 2 groups in this particular analysis with all but one specimen (NHMUK PV P3578b) of M. pusillus falling within the same space as the specimens of M. peachi. [file peerj-03-1331-s002.png]

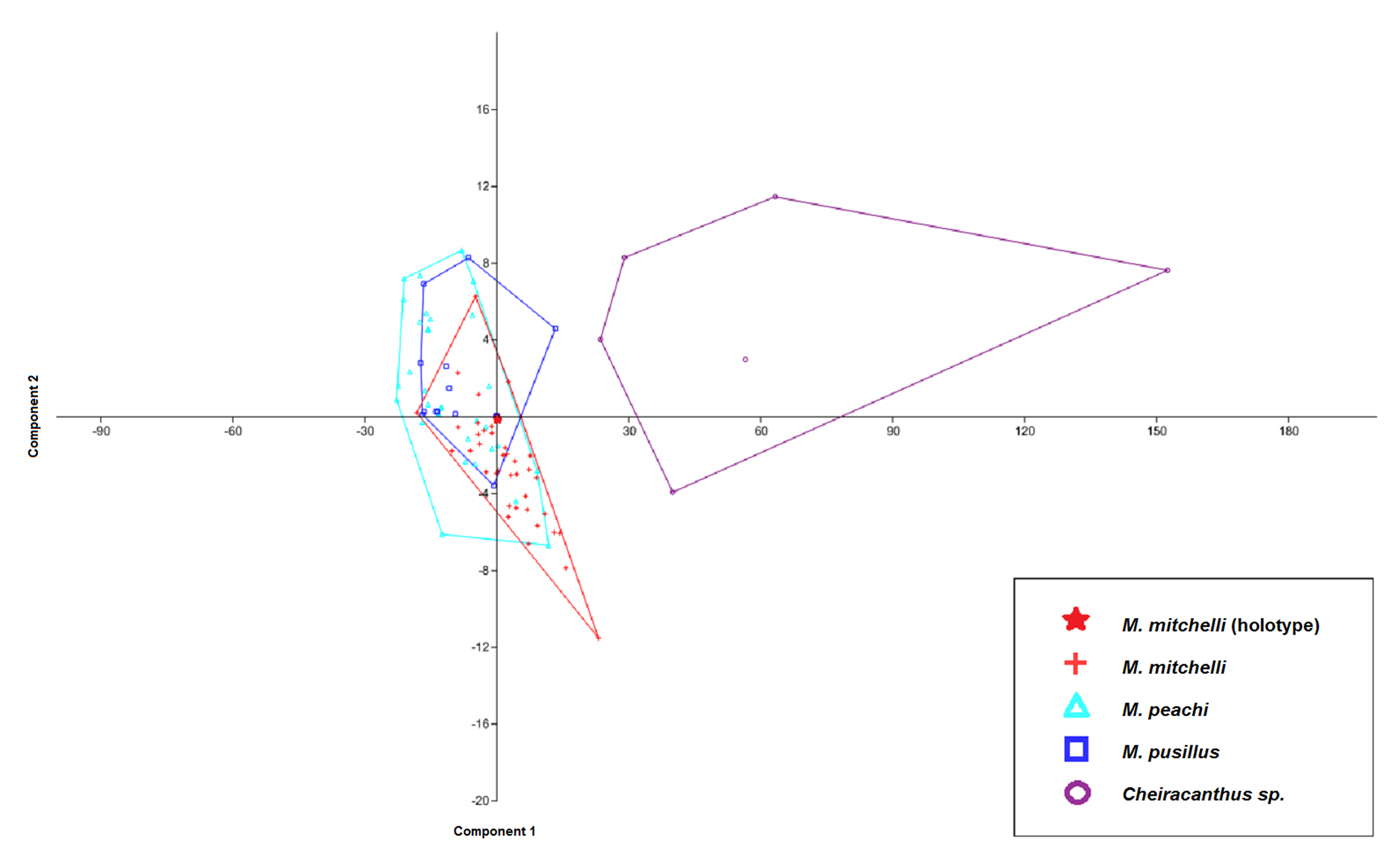

Supplement: SOM S3 — As in Fig. 4, this figure shows how this analysis produced a great degree of sepeartionbetween specimens of Mesacanthus and Cheiracanthus. Again, this lends evidence to thestrength of this method and its potential for use in future studies of other similar taxa. [file peerj-03-1331-s003.png]

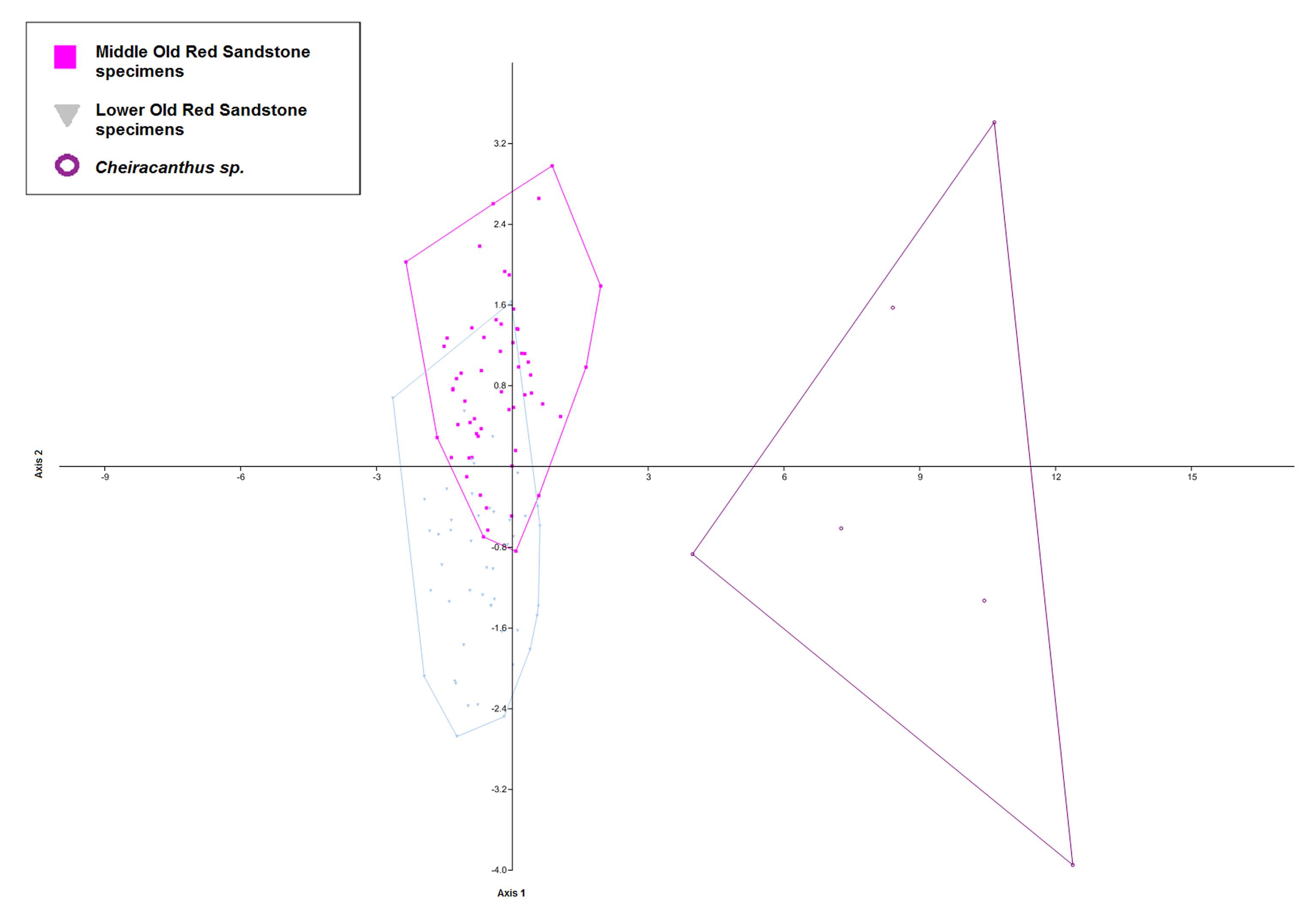

Supplement: SOM S4 — The figure shows the CVA scatter that is produced when the specimens of Mesacanthus are divided into 2 groups (Middle Devonian specimens and Lower Devonian specimens) and analysed alongside specimens ofCheiracanthus. The confusion matrix produced in this analysis and the uncorrected and Bonferonni corrected Hotelling’s p-values are shown in Table 6. [file peerj-03-1331-s004.png]

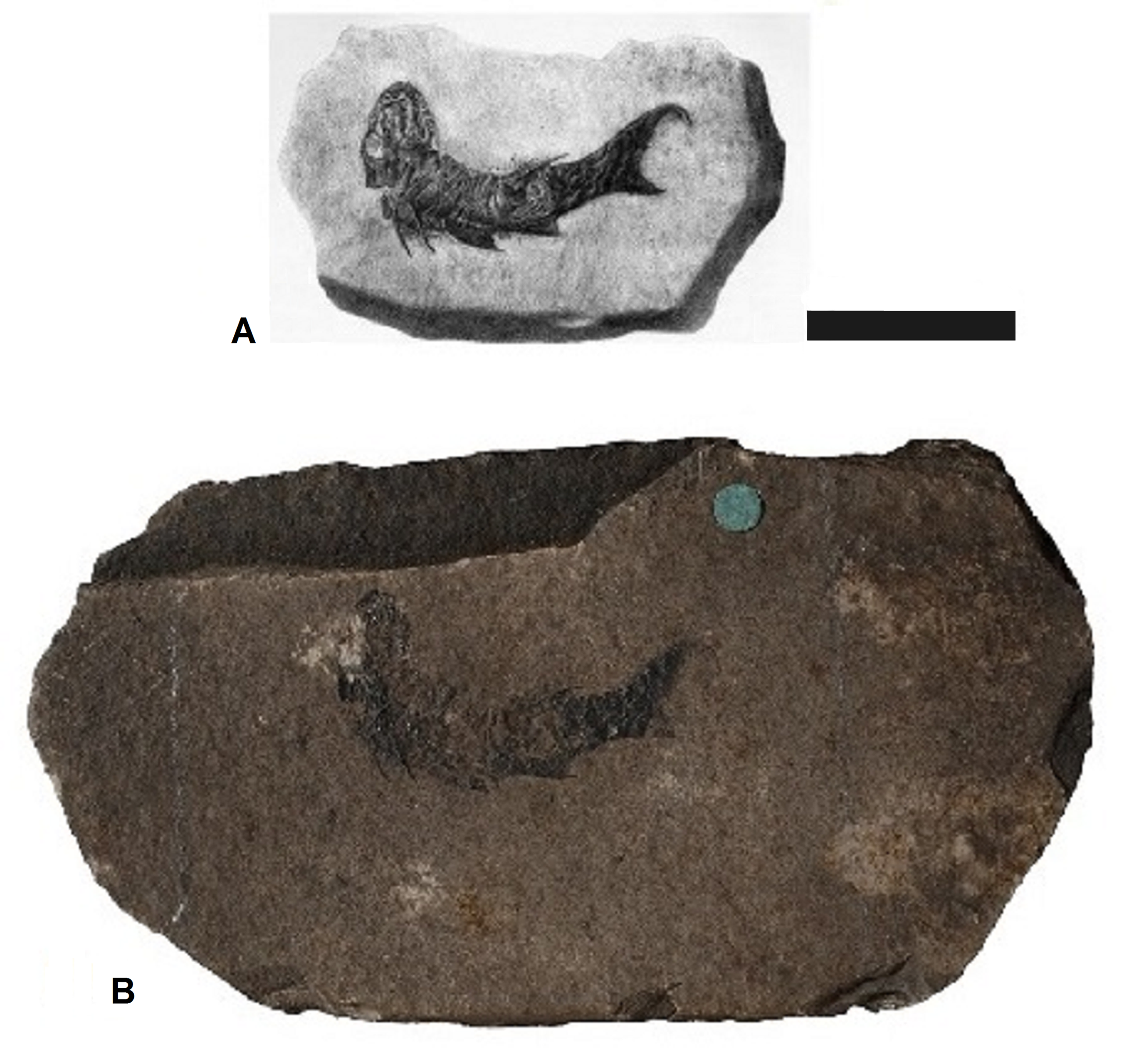

Supplement: SOM S6 — (A) Figure from Egerton (1861, pl. 6 Fig. 1) which is the only figure given with the original description of M. peachi. (B) Specimen GSM 21448, which is very likely the specimen figured by Egerton (1861) despite the disparity in the size of the slab it appears on and of the slab depicted in the illustration. Scale bar = 20 mm. [file peerj-03-1331-s006.png]

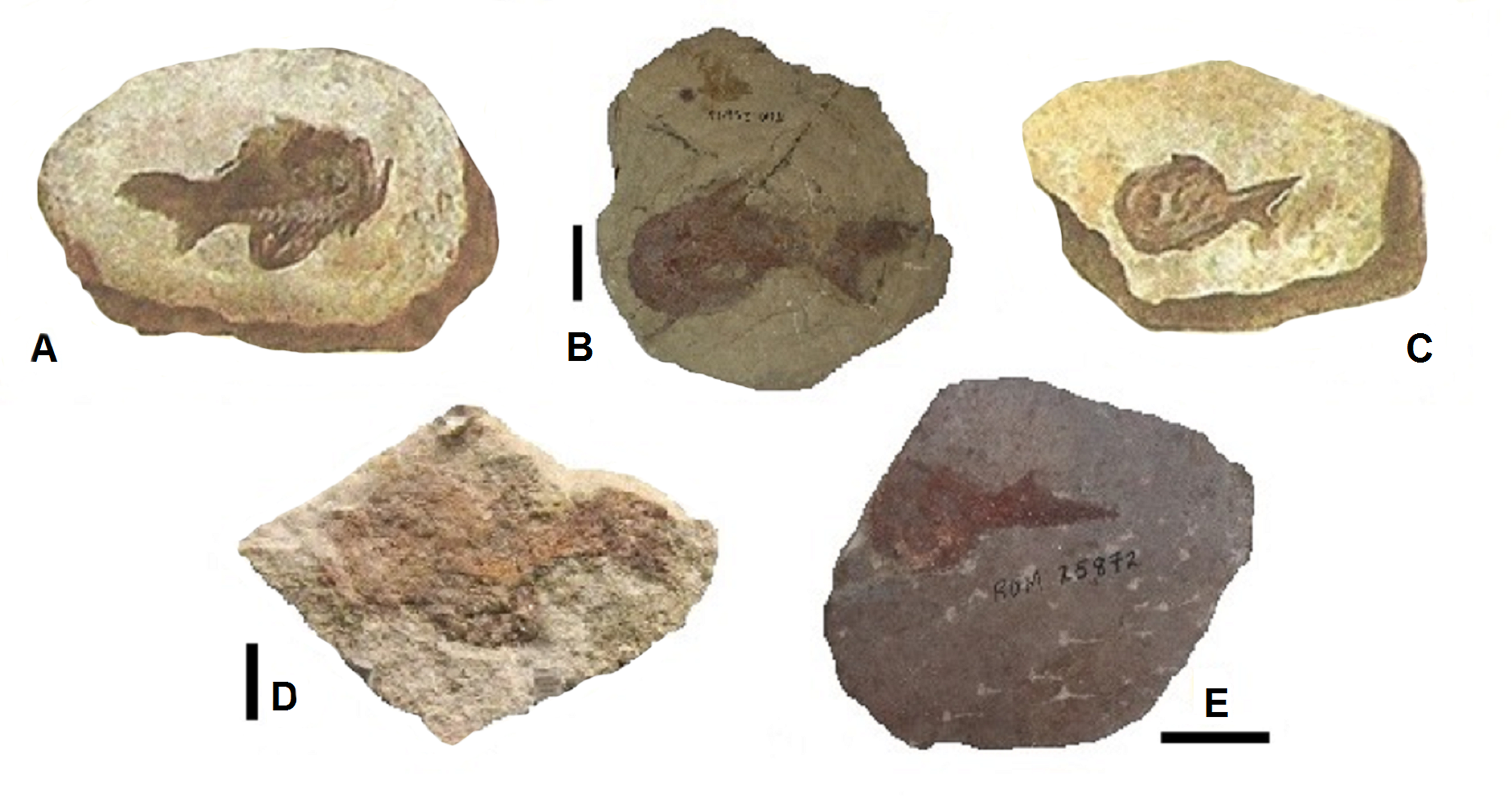

Supplement: SOM S7 — (A) One of three figures given by Agassiz (1844–1845, pl. 28, Figs. 8–10) with the original description of M. pusillus. (B) Specimen ROM 25846, which is possibly one of the specimens (or counterpart to) that was figured by Agassiz (1844–1845). (C) Another of the three figuresgiven by Agassiz (1844–1845). (D) Specimen ELGNM 1978.191.1, which could also be one of the specimens (or counter part to) that was figured by Agassiz (1844–1845). (E) Specimen ROM 25872, which could also be one of the specimens (or counter part to) that was figured by Agassiz (1844–1845). Scale bar = 20mm. [file peerj-03-1331-s007.png]
