# Supplementary material for: An investigation of the genus Mesacanthus (Chordata: Acanthodii) from the Orcadian Basin and Midland Valley areas of Northern and Central Scotland using traditional morphometrics
Source: PeerJ. 2015 Oct 29;3:e1331. doi: 10.7717/peerj.1331 (PMC4631467; doi:10.7717/peerj.1331)
Supplement: SOM S5 — The confusion matrix shows the relatively low amount of confusion between specimens from the 3 groups in this particular analysis. The uncorrected and Bonferroni corrected Hotelling’s p-values show how each of these groups was found to be significantly different from the others. This is important because this shows that when M. peachi and M. pusillus are synonymised the single taxon is found to still be significantly different from other acanthodian taxa. Red = not significant; Green = significant. [file peerj-03-1331-s005.docx]

|  |  | ***Mesacanthus*** | |  |  |
| --- | --- | --- | --- | --- | --- |
|  |  | **Middle Devonian** | **Lower Devonian** | ***Cheiracanthus*** | **Total** |
| **Confusion matrix** | **Middle Devonian** | 47 | 11 | 0 | 58 |
|  | **Lower Devonian** | 10 | 38 | 0 | 48 |
|  | **Cheiracanthus** | 2 | 0 | 4 | 6 |
|  | **Total** | 59 | 49 | 4 | 112 |
|  |  |  |  |  |  |
|  |  | ***Mesacanthus*** | |  |  |
|  |  | **Middle Devonian** | **Lower Devonian** | ***Cheiracanthus*** |  |
| **Uncorrected** | **Middle Devonian** | - |  |  |  |
|  | **Lower Devonian** | 4.01E-08 | - |  |  |
|  | **Cheiracanthus** | 7.96E-20 | 2.57E-17 | - |  |
|  |  |  |  |  |  |
|  |  | ***Mesacanthus*** | |  |  |
|  |  | **Middle Devonian** | **Lower Devonian** | ***Cheiracanthus*** |  |
| **Bonferonni corrected** | **Middle Devonian** | - |  |  |  |
|  | **Lower Devonian** | 1.34E-08 | - |  |  |
|  | **Cheiracanthus** | 2.65E-20 | 8.57E-18 | - |  |
