# Supplementary material for: An investigation of the genus Mesacanthus (Chordata: Acanthodii) from the Orcadian Basin and Midland Valley areas of Northern and Central Scotland using traditional morphometrics
Source: PeerJ. 2015 Oct 29;3:e1331. doi: 10.7717/peerj.1331 (PMC4631467; doi:10.7717/peerj.1331)
Supplement: SOM S8 [file peerj-03-1331-s008.docx]

| **All *Mesacanthus* species** | | | **Middle Devonian species** | | | ***Mesacanthus* and *Cheircanthus*** | | |
| --- | --- | --- | --- | --- | --- | --- | --- | --- |
| **PC** | **Eigenvalue** | **% variance** | **PC** | **Eigenvalue** | **% variance** | **PC** | **Eigenvalue** | **% variance** |
| 1 | 92.335 | 83.356 | 1 | 58.7075 | 79.608 | 1 | 395.307 | 91.804 |
| 2 | 5.62545 | 5.0784 | 2 | 7.33653 | 9.9484 | 2 | 14.8331 | 3.4447 |
| 3 | 4.18922 | 3.7818 | 3 | 3.01642 | 4.0903 | 3 | 5.72834 | 1.3303 |
| 4 | 2.78259 | 2.512 | 4 | 1.79586 | 2.4352 | 4 | 4.47808 | 1.04 |
| 5 | 2.14936 | 1.9403 | 5 | 0.852459 | 1.1559 | 5 | 3.75927 | 0.87303 |
| 6 | 1.10194 | 0.99478 | 6 | 0.707207 | 0.95898 | 6 | 2.33729 | 0.5428 |
| 7 | 0.965336 | 0.87146 | 7 | 0.517415 | 0.70162 | 7 | 1.39219 | 0.32331 |
| 8 | 0.666164 | 0.60138 | 8 | 0.439179 | 0.59553 | 8 | 1.2211 | 0.28358 |
| 9 | 0.51535 | 0.46523 | 9 | 0.252962 | 0.34302 | 9 | 0.852287 | 0.19793 |
| 10 | 0.442111 | 0.39912 | 10 | 0.120237 | 0.16304 | 10 | 0.691704 | 0.16064 |
