# Supplementary material for: An investigation of the genus Mesacanthus (Chordata: Acanthodii) from the Orcadian Basin and Midland Valley areas of Northern and Central Scotland using traditional morphometrics
Source: PeerJ. 2015 Oct 29;3:e1331. doi: 10.7717/peerj.1331 (PMC4631467; doi:10.7717/peerj.1331)
Supplement: Supplemental Information 1 — The table records all of the measurements taken for each specimen looked at in this study. [file peerj-03-1331-s009.docx]

| **Taxon** | **Specimen number** | **L** | **W** | **PP** | **PA** |
| --- | --- | --- | --- | --- | --- |
| M. peachi | ABDUG Pal.10885 | 27.0 | 5.5 | ? | ? |
| M. peachi | ABDUG Pal.10886 | 23.0 | 5.0 | ? | ? |
| M. peachi | GLAHM V7019a | 25.0 | 6.5 | 4.3 | 5.6 |
| M. peachi | GLAHM V7019b | 26.2 | 6.0 | ? | ? |
| M. peachi | GLAHM V7396 | 46.2 | 11.1 | ? | ? |
| M. peachi | GLAHM V7418 | 26.1 | 5.1 | ? | ? |
| M. peachi | ABDUG Pal.10887 | 18.0 | 4.0 | ? | ? |
| M. peachi | NHMUK PV P61714 | 38.5 | 8.5 | 5.5 | 5.7 |
| M. peachi | NHMUK PV P22201 | 30.9 | 5.6 | ? | ? |
| M. peachi | NHMUK PV P38583 | 26.1 | 5.1 | ? | ? |
| M. peachi | NHMUK PV P43967 | 42.3 | 8.5 | 11.4 | 8.4 |
| M. peachi | NHMUK PV P49668 | 37.9 | 8.5 | 7.3 | 6.0 |
| M. peachi | NHMUK PV P49669 | 41.6 | 7.1 | 8.0 | 6.3 |
| M. peachi | NHMUK PV P61673 | 29.1 | 6.8 | ? | ? |
| M. peachi | NHMUK PV P61674 | 25.6 | 4.2 | ? | ? |
| M. peachi | NHMUK PV P61679a | 19.1 | 3.2 | 4.7 | 3.0 |
| M. peachi | NHMUK PV P61679b | 21.3 | 2.9 | ? | ? |
| M. peachi | NHMUK PV P61680 | 23.9 | 3.9 | 4.6 | 4.5 |
| M. peachi | NHMUK PV P61682 | 25.9 | 6.4 | 5.7 | 4.8 |
| M. peachi | NHMUK PV P61683 | 30.1 | 6.0 | ? | ? |
| M. peachi | NHMUK PV P61699 | 31.0 | 6.2 | ? | ? |
| M. peachi | NHMUK PV P61700 | 20.2 | 4.9 | ? | ? |
| M. peachi | NHMUK PV P61701 | 28.9 | 5.0 | 6.0 | ? |
| M. peachi | NHMUK PV P61704 | 27.6 | 6.1 | ? | 4.8 |
| M. peachi | NHMUK PV P61705 | 19.1 | 3.0 | ? | ? |
| M. peachi | NHMUK PV P61714 | 32.1 | 6.1 | 7.2 | 5.3 |
| M. peachi | NHMUK PV P61715 | 44.3 | 6.7 | 6.2 | 12.6 |
| M. peachi | NHMUK PV P61744 | 16.3 | 6.6 | ? | ? |
| M. peachi | NHMUK PV P61766 | 49.2 | 9.8 | ? | 7.6 |
| M. peachi | NHMUK PV P61768 | 24.8 | 4.8 | ? | ? |
| M. peachi | ABDUG Pal.D14 | 25.0 | 8.0 | ? | ? |
| M. peachi | ABDUG Pal.D92 | 60.0 | 17.6 | ? | ? |
| M. peachi | ABDUG Pal.D91 | 52.0 | 15.0 | 15.0 | 11.0 |
| M. peachi | ABDUG Pal.JA4 | 17.5 | 5.0 | 3.0 | 4.5 |
| M. peachi | ABDUG Pal.D56 | 50.0 | 12.5 | 10.5 | 8.0 |
| M. peachi | ABDUG Pal.JA1 | 28.0 | 7.0 | 8.0 | 4.0 |
| M. peachi | ABDUG Pal.D93b | 32.0 | 8.0 | 5.5 | 3.0 |
| M. peachi | ABDUG Pal.D90 | 29.5 | 7.3 | 7.5 | 2.5 |
| M. peachi | ABDUG Pal.D93i | 35.0 | 8.4 | 7.5 | 5.5 |
| M. peachi | ABDUG Pal.D51 | 52.0 | 12.0 | 15.0 | 8.0 |
| M. peachi | ABDUG Pal.D214 | 37.0 | 8.5 | ? | ? |
|  |  |  |  |  |  |
| M. pusillus | ABDUG Pal.D54 | 27.0 | 6.0 | 5.5 | 4.5 |
| M. pusillus | ABDUG Pal.D13 | 28.0 | 6.0 | 8.5 | 4.0 |
| M. pusillus | ABDUG Pal. D55 | 28.5 | 6.0 | 4.5 | 7.5 |
| M. pusillus | ABDUG Pal.JA2 | 30.0 | 6.3 | 7.0 | 3.5 |
| M. pusillus | ABDUG Pal.D52 | 38.5 | 8.0 | 7.5 | 6.5 |
| M. pusillus | ABDUG Pal.JA3 | 32.0 | 6.5 | 6.0 | 5.0 |
| M. pusillus | ABDUG Pal.D53 | 44.5 | 9.0 | ? | ? |
| M. pusillus | GLAHM V3573 | 32.6 | 6.5 | 9.3 | 2.8 |
| M. pusillus | NHMUK PV P1329a | 31.0 | 6.4 | ? | ? |
| M. pusillus | NHMUK PV P1329b | 43.7 | 9.2 | ? | ? |
| M. pusillus | GLAHM V2361 | 26.0 | 4.6 | ? | ? |
| M. pusillus | NHMUK PV P3578b | 57.7 | 12.3 | 10.1 | 14.6 |
| M. pusillus | NHMUK PV P9771 | 44.4 | 7.1 | ? | ? |
| M. pusillus | NHMUK PV P35784 | 45.0 | 7.0 | 5.6 | 10.2 |
| M. pusillus | NHMUK PV P40077 | 24.6 | 6.0 | ? | ? |
| M. pusillus | NHMUK PV P40078 | 32.4 | 5.5 | ? | ? |
| M. pusillus | NHMUK PV P43019 | 31.7 | 5.2 | ? | ? |
|  |  |  |  |  |  |
| M. mitchelli | ABDUG Pal.D171 | 37.5 | 6.0 | 7.0 | 6.5 |
| M. mitchelli | ABDUG Pal.D172 | 40.0 | 6.0 | 7.0 | 7.0 |
| M. mitchelli | ABDUG Pal.D173 | 58.5 | 11.0 | 14.0 | 6.5 |
| M. mitchelli | NHMUK PV P1331 | 60.2 | 9.7 | 8.9 | 7.0 |
| M. mitchelli | ABDUG Pal.12331a | 49.0 | 7.5 | ? | ? |
| M. mitchelli | ABDUG Pal.12229 | 64.0 | 9.0 | 13.0 | 8.5 |
| M. mitchelli | ABDUG Pal.12236a | 36.0 | 6.0 | ? | ? |
| M. mitchelli | ABDUG Pal.12236b | 31.8 | 5.8 | ? | ? |
| M. mitchelli | ABDUG Pal.12236c | 33.0 | 4.5 | ? | ? |
| M. mitchelli | ABDUG Pal.12236d | 34.0 | 4.5 | ? | ? |
| M. mitchelli | ABDUG Pal.12236f | 33.0 | 6.0 | ? | ? |
| M. mitchelli | ABDUG Pal.12236h | 28.0 | 4.0 | ? | ? |
| M. mitchelli | ABDUG Pal.12236i | 24.0 | 5.0 | ? | ? |
| M. mitchelli | NMS G. 3053 ^ | 68.0 | 13.0 | 10.5 | 6.0 |
| M. mitchelli | NMS G. 160 ^ | 61.0 | 10.0 | 14.0 | 6.5 |
| M. mitchelli | NMS G. 3054 ^ | 52.0 | 9.0 | 8.0 | 7.5 |
| M. mitchelli | NMS G. 46. 5 ^ | 54.0 | 7.0 | 11.0 | 7.0 |
| M. mitchelli | GLAHM V8118 b | 28.6 | 5.7 | ? | ? |
| M. mitchelli | GLAHM V8118 c | 47.2 | 7.9 | 5.7 | 10.7 |
| M. mitchelli | GLAHM V8118 d | 55.0 | 11.4 | 10.7 | 8.5 |
| M. mitchelli | NHMUK PV P9204a | 64.9 | 10.3 | 11.2 | 8.9 |
| M. mitchelli | NHMUK PV P4005 | 57.6 | 9.2 | 8.2 | 13.1 |
| M. mitchelli | NMS G. 62 2 ^ | 53.5 | 9.5 | 9.5 | 7.0 |
| M. mitchelli | NMS G. 62 3 ^ | 47.5 | 9.5 | 11.5 | 6.0 |
| M. mitchelli | NMS G. 62 4 ^ | 55.0 | 11.0 | 14.5 | 8.0 |
| M. mitchelli | GLAHM V3522 | 66.5 | 11.6 | 10.0 | 8.9 |
| M. mitchelli | GLAHM V3524 | 66.8 | 14.0 | 10.3 | 8.4 |
| M. mitchelli | NMS G. 1891 92. 306 | 46.5 | 9.0 | 11.0 | 7.0 |
| M. mitchelli | NMS G. 1891 92. 301 | 54.0 | 7.5 | 10.5 | 7.5 |
| M. mitchelli | NMS G. 1891 92. 300 | 48.0 | 7.0 | 11.0 | 7.0 |
| M. mitchelli | NMS G. 1891 92. 299 | 73.0 | 15.0 | ? | ? |
| M. mitchelli | NMS G. 1891 92. 309 | 49.0 | 9.0 | 10.5 | 7.0 |
| M. mitchelli | NHMUK PV P560* | 50.9 | 8.0 | 11.7 | 8.7 |
| M. mitchelli | NHMUK PV P1330 | 39.2 | 7.4 | 9.0 | 5.6 |
| M. mitchelli | NHMUK PV P4004 | 38.0 | 6.7 | 10.7 | 7.8 |
| M. mitchelli | NMS G. 1887 35. 1F | 71.0 | 12.0 | 11.0 | 9.0 |
| M. mitchelli | NMS G. 1890 6. 28 | 51.0 | 8.0 | ? | ? |
| M. mitchelli | NMS G. 1891 92. 276 | 53.0 | 8.5 | 12.0 | 6.0 |
| M. mitchelli | NMS G. 1891 92. 278 | 55.0 | 11.0 | 13.0 | 5.0 |
| M. mitchelli | NMS G. 1887 35. 1B | 50.0 | 10.0 | 10.0 | 8.0 |
| M. mitchelli | NMS G. 1891 92. 283 | 57.0 | 10.0 | 12.5 | 8.0 |
| M. mitchelli | NMS G. 1891 92. 287 | 61.0 | 9.0 | ? | ? |
| M. mitchelli | NMS G. 1891 92. 286 | 45.0 | 8.0 | 10.0 | 7.0 |
| M. mitchelli | NMS G. 1891 92. 281 | 68.0 | 14.0 | 17.0 | 9.0 |
| M. mitchelli | NMS G. 1891 92. 285 | 50.0 | 10.0 | ? | 10.0 |
| M. mitchelli | NMS G. 1891 92. 291 | 25.0 | 4.0 | 6.0 | 3.5 |
| M. mitchelli | NMS G. 1891 92. 280 | 41.0 | 7.0 | 7.0 | 4.0 |
| M. mitchelli | GLAHM V3521 | 32.0 | 5.2 | 4.6 | 11.6 |
|  |  |  |  |  |  |
|  |  |  |  |  |  |
| Cheiracanthus sp. | ABDUG Pal.D68 | 245.0 | 56.0 | 47.0 | 37.0 |
| Cheiracanthus sp. | ABDUG Pal.D166 | 95.0 | 37.0 | 35.0 | 26.0 |
| Cheiracanthus sp. | ABDUG Pal.D209 | 100.0 | 26.0 | 20.0 | 11.0 |
| Cheiracanthus sp. | ABDUG Pal.D210 | 104.0 | 26.0 | 13.0 | 23.0 |
| Cheiracanthus sp. | ABDUG Pal.D211 | 102.0 | 28.0 | 25.0 | 26.0 |
| Cheiracanthus sp. | ABDUG Pal.D212 | 95.0 | 21.0 | 16.0 | 17.5 |

| **Taxon** | **Specimen number** | **LD** | **LA** | **A_D** | **lPCF** |
| --- | --- | --- | --- | --- | --- |
| M. peachi | ABDUG Pal.10885 | ? | ? | ? | ? |
| M. peachi | ABDUG Pal.10886 | ? | ? | ? | ? |
| M. peachi | GLAHM V7019a | 19.8 | 16.6 | 3.2 | ? |
| M. peachi | GLAHM V7019b | 13.3 | 12.5 | 0.8 | ? |
| M. peachi | GLAHM V7396 | ? | ? | ? | ? |
| M. peachi | GLAHM V7418 | 12.0 | 9.4 | 2.6 | ? |
| M. peachi | ABDUG Pal.10887 | ? | ? | ? | ? |
| M. peachi | NHMUK PV P61714 | 23.7 | 22.4 | 1.3 | ? |
| M. peachi | NHMUK PV P22201 | ? | ? | ? | ? |
| M. peachi | NHMUK PV P38583 | 13.4 | ? | ? | 4.8 |
| M. peachi | NHMUK PV P43967 | 25.0 | 22.1 | 2.9 | 4.8 |
| M. peachi | NHMUK PV P49668 | 23.5 | 22.1 | 1.4 | ? |
| M. peachi | NHMUK PV P49669 | 25.1 | 23.6 | 1.5 | 4.8 |
| M. peachi | NHMUK PV P61673 | 16.1 | 14.6 | 1.5 | ? |
| M. peachi | NHMUK PV P61674 | ? | ? | ? | ? |
| M. peachi | NHMUK PV P61679a | 13.3 | 10.5 | 2.8 | 1.9 |
| M. peachi | NHMUK PV P61679b | 12.1 | 10.4 | 1.7 | ? |
| M. peachi | NHMUK PV P61680 | 13.8 | 13.1 | 0.7 | ? |
| M. peachi | NHMUK PV P61682 | ? | 12.7 | ? | ? |
| M. peachi | NHMUK PV P61683 | 18.1 | ? | ? | ? |
| M. peachi | NHMUK PV P61699 | ? | ? | ? | ? |
| M. peachi | NHMUK PV P61700 | 15.1 | 14.5 | 0.6 | ? |
| M. peachi | NHMUK PV P61701 | ? | ? | ? | ? |
| M. peachi | NHMUK PV P61704 | 15.6 | 14.2 | 1.4 | ? |
| M. peachi | NHMUK PV P61705 | ? | ? | ? | ? |
| M. peachi | NHMUK PV P61714 | 16.3 | 15.7 | 0.6 | 3.1 |
| M. peachi | NHMUK PV P61715 | 25.8 | 24.9 | 0.9 | ? |
| M. peachi | NHMUK PV P61744 | 16.3 | 14.1 | 2.2 | ? |
| M. peachi | NHMUK PV P61766 | 28.0 | 26.1 | 1.9 | ? |
| M. peachi | NHMUK PV P61768 | 14.3 | 14.0 | 0.3 | ? |
| M. peachi | ABDUG Pal.D14 | 17.0 | ? | ? | ? |
| M. peachi | ABDUG Pal.D92 | 38.0 | 34.0 | 4.0 | ? |
| M. peachi | ABDUG Pal.D91 | 34.5 | 32.0 | 2.5 | ? |
| M. peachi | ABDUG Pal.JA4 | 12.5 | 12.0 | 0.5 | 4.0 |
| M. peachi | ABDUG Pal.D56 | 32.0 | 29.5 | 2.5 | 3.0 |
| M. peachi | ABDUG Pal.JA1 | 17.5 | 15.5 | 2.0 | 4.0 |
| M. peachi | ABDUG Pal.D93b | 19.0 | 17.0 | 2.0 | ? |
| M. peachi | ABDUG Pal.D90 | 17.0 | 15.0 | 2.0 | 3.0 |
| M. peachi | ABDUG Pal.D93i | 19.0 | 18.0 | 1.0 | ? |
| M. peachi | ABDUG Pal.D51 | 26.0 | 23.0 | 3.0 | ? |
| M. peachi | ABDUG Pal.D214 | ? | ? | ? | ? |
|  |  |  |  |  |  |
| M. pusillus | ABDUG Pal.D54 | 15.5 | 13.5 | 2.0 | ? |
| M. pusillus | ABDUG Pal.D13 | 18.5 | 18.0 | 0.5 | ? |
| M. pusillus | ABDUG Pal. D55 | 18.5 | 17.5 | 1.0 | 2.5 |
| M. pusillus | ABDUG Pal.JA2 | 16.5 | 16.0 | 0.5 | 4.0 |
| M. pusillus | ABDUG Pal.D52 | 19.0 | 20.0 | 1.0 | ? |
| M. pusillus | ABDUG Pal.JA3 | 17.0 | 15.0 | 2.0 | 3.5 |
| M. pusillus | ABDUG Pal.D53 | ? | ? | ? | ? |
| M. pusillus | GLAHM V3573 | 20.9 | 20.0 | 0.9 | ? |
| M. pusillus | NHMUK PV P1329a | ? | ? | ? | ? |
| M. pusillus | NHMUK PV P1329b | ? | ? | ? | ? |
| M. pusillus | GLAHM V2361 | 15.0 | ? | ? | ? |
| M. pusillus | NHMUK PV P3578b | ? | 43.4 | ? | ? |
| M. pusillus | NHMUK PV P9771 | ? | ? | ? | ? |
| M. pusillus | NHMUK PV P35784 | 28.4 | 26.8 | 1.6 | ? |
| M. pusillus | NHMUK PV P40077 | 14.1 | 13.1 | 1.0 | ? |
| M. pusillus | NHMUK PV P40078 | ? | ? | ? | ? |
| M. pusillus | NHMUK PV P43019 | 18.7 | 18.5 | 0.2 | 4.0 |
|  |  |  |  |  |  |
| M. mitchelli | ABDUG Pal.D171 | 22.0 | 20.5 | 1.5 | 6.0 |
| M. mitchelli | ABDUG Pal.D172 | 25.0 | 23.0 | 2.0 | 7.5 |
| M. mitchelli | ABDUG Pal.D173 | 29.5 | 26.5 | 3.0 | 6.0 |
| M. mitchelli | NHMUK PV P1331 | 31.6 | 27.8 | 3.8 | 6.1 |
| M. mitchelli | ABDUG Pal.12331a | ? | ? | ? | ? |
| M. mitchelli | ABDUG Pal.12229 | 34.0 | 32.0 | 2.0 | 5.5 |
| M. mitchelli | ABDUG Pal.12236a | ? | ? | ? | ? |
| M. mitchelli | ABDUG Pal.12236b | ? | ? | ? | ? |
| M. mitchelli | ABDUG Pal.12236c | ? | ? | ? | ? |
| M. mitchelli | ABDUG Pal.12236d | ? | ? | ? | ? |
| M. mitchelli | ABDUG Pal.12236f | ? | ? | ? | ? |
| M. mitchelli | ABDUG Pal.12236h | ? | ? | ? | ? |
| M. mitchelli | ABDUG Pal.12236i | ? | ? | ? | ? |
| M. mitchelli | NMS G. ‘3053’ | 33.0 | 28.0 | 5.0 | 7.5 |
| M. mitchelli | NMS G. ‘160’ | 34.0 | 30.5 | 3.5 | 4.0 |
| M. mitchelli | NMS G. ‘3054’ | 28.0 | 27.0 | 1.0 | 4.0 |
| M. mitchelli | NMS G. ‘46. 5’ | 30.0 | 29.0 | 1.0 | ? |
| M. mitchelli | GLAHM V8118 b | 17.9 | ? | ? | ? |
| M. mitchelli | GLAHM V8118 c | 25.7 | 24.3 | 1.4 | ? |
| M. mitchelli | GLAHM V8118 d | 33.6 | 30.0 | 3.6 | ? |
| M. mitchelli | NHMUK PV P9204a | 40.1 | 38.0 | 2.1 | ? |
| M. mitchelli | NHMUK PV P4005 | 32.9 | 31.6 | 1.3 | 7.4 |
| M. mitchelli | NMS G. ‘62 2’ | 29.5 | 27.0 | 2.5 | 6.0 |
| M. mitchelli | NMS G. ‘62 3’ | 29.0 | 26.5 | 2.5 | 6.0 |
| M. mitchelli | NMS G. ‘62 4’ | 39.0 | 35.5 | 3.5 | 5.5 |
| M. mitchelli | GLAHM V3522 | 36.2 | 33.5 | 2.7 | 6.2 |
| M. mitchelli | GLAHM V3524 | 36.1 | 32.0 | 4.1 | ? |
| M. mitchelli | NMS G. 1891 92. 306 | 27.0 | 25.0 | 2.0 | 5.5 |
| M. mitchelli | NMS G. 1891 92. 301 | 27.0 | 24.0 | 3.0 | 5.0 |
| M. mitchelli | NMS G. 1891 92. 300 | 25.5 | 23.5 | 2.0 | 4.0 |
| M. mitchelli | NMS G. 1891 92. 299 | 46.0 | 44.0 | 2.0 | ? |
| M. mitchelli | NMS G. 1891 92. 309 | 32.0 | 27.5 | 4.5 | 4.0 |
| M. mitchelli | NHMUK PV P560* | 31.3 | 29.2 | 2.1 | 4.5 |
| M. mitchelli | NHMUK PV P1330 | 24.4 | 23.2 | 1.2 | 7.4 |
| M. mitchelli | NHMUK PV P4004 | 21.3 | 18.5 | 2.8 | 6.7 |
| M. mitchelli | NMS G. 1887 35. 1F | 38.0 | 35.0 | 3.0 | ? |
| M. mitchelli | NMS G. 1890 6. 28 | ? | ? | ? | ? |
| M. mitchelli | NMS G. 1891 92. 276 | 29.0 | 26.0 | 3.0 | ? |
| M. mitchelli | NMS G. 1891 92. 278 | 35.0 | 31.0 | 4.0 | 4.0 |
| M. mitchelli | NMS G. 1887 35. 1B | 32.0 | 26.5 | 5.5 | 5.5 |
| M. mitchelli | NMS G. 1891 92. 283 | 34.0 | 28.0 | 6.0 | ? |
| M. mitchelli | NMS G. 1891 92. 287 | ? | ? | ? | ? |
| M. mitchelli | NMS G. 1891 92. 286 | 27.0 | 25.0 | 2.0 | 3.0 |
| M. mitchelli | NMS G. 1891 92. 281 | 28.5 | 25.5 | 3.0 | 5.0 |
| M. mitchelli | NMS G. 1891 92. 285 | 25.0 | 21.5 | 3.5 | ? |
| M. mitchelli | NMS G. 1891 92. 291 | 16.0 | 14.0 | 2.0 | 2.0 |
| M. mitchelli | NMS G. 1891 92. 280 | 22.0 | 19.0 | 3.0 | 3.0 |
| M. mitchelli | GLAHM V3521 | ? | 18.9 | ? | ? |
|  |  |  |  |  |  |
|  |  |  |  |  |  |
| Cheiracanthus sp. | ABDUG Pal.D68 | 103.0 | 137.0 | 34.0 | 16.0 |
| Cheiracanthus sp. | ABDUG Pal.D166 | 55.0 | 70.0 | 15.0 | 15.0 |
| Cheiracanthus sp. | ABDUG Pal.D209 | 51.0 | 55.0 | 4.0 | 17.0 |
| Cheiracanthus sp. | ABDUG Pal.D210 | 37.0 | 48.0 | 11.0 | 11.0 |
| Cheiracanthus sp. | ABDUG Pal.D211 | 59.0 | 62.0 | 13.0 | 20.5 |
| Cheiracanthus sp. | ABDUG Pal.D212 | 37.0 | 43.5 | 6.5 | 8.0 |

| **Taxon** | **Specimen number** | **lPLF** | **lAF** | **lDF** | **L/W** |
| --- | --- | --- | --- | --- | --- |
| M. peachi | ABDUG Pal.10885 | ? | ? | ? | 4.909091 |
| M. peachi | ABDUG Pal.10886 | ? | ? | ? | 4.6 |
| M. peachi | GLAHM V7019a | ? | ? | ? | 3.846154 |
| M. peachi | GLAHM V7019b | ? | ? | ? | 4.366667 |
| M. peachi | GLAHM V7396 | ? | ? | ? | 4.162162 |
| M. peachi | GLAHM V7418 | ? | 3.4 | 3.4 | 5.117647 |
| M. peachi | ABDUG Pal.10887 | ? | ? | ? | 4.5 |
| M. peachi | NHMUK PV P61714 | 5 | 4.7 | 3.9 | 4.529412 |
| M. peachi | NHMUK PV P22201 | ? | ? | ? | 5.517857 |
| M. peachi | NHMUK PV P38583 | ? | ? | 3.4 | 5.117647 |
| M. peachi | NHMUK PV P43967 | 2.5 | 3.6 | 5.7 | 4.976471 |
| M. peachi | NHMUK PV P49668 | 4.7 | 3.8 | 6.4 | 4.458824 |
| M. peachi | NHMUK PV P49669 | ? | 2.9 | 4.5 | 5.859155 |
| M. peachi | NHMUK PV P61673 | ? | ? | 2.8 | 4.279412 |
| M. peachi | NHMUK PV P61674 | ? | ? | ? | 6.095238 |
| M. peachi | NHMUK PV P61679a | ? | 1.7 | 2.2 | 5.96875 |
| M. peachi | NHMUK PV P61679b | ? | 1.3 | 1.5 | 7.344828 |
| M. peachi | NHMUK PV P61680 | ? | ? | 1.9 | 6.128205 |
| M. peachi | NHMUK PV P61682 | 1.9 | 1.7 | ? | 4.046875 |
| M. peachi | NHMUK PV P61683 | ? | ? | 2.1 | 5.016667 |
| M. peachi | NHMUK PV P61699 | ? | ? | ? | 5 |
| M. peachi | NHMUK PV P61700 | ? | ? | 1.9 | 4.122449 |
| M. peachi | NHMUK PV P61701 | ? | ? | ? | 5.78 |
| M. peachi | NHMUK PV P61704 | ? | ? | ? | 4.52459 |
| M. peachi | NHMUK PV P61705 | ? | ? | ? | 6.366667 |
| M. peachi | NHMUK PV P61714 | 3.3 | 3.5 | 4.1 | 5.236542 |
| M. peachi | NHMUK PV P61715 | 3.6 | 5.1 | 5.4 | 6.61194 |
| M. peachi | NHMUK PV P61744 | ? | 2.6 | 2.5 | 2.469697 |
| M. peachi | NHMUK PV P61766 | ? | ? | ? | 5.020408 |
| M. peachi | NHMUK PV P61768 | ? | 1.9 | 1.5 | 5.166667 |
| M. peachi | ABDUG Pal.D14 | ? | ? | ? | 3.125 |
| M. peachi | ABDUG Pal.D92 | ? | ? | ? | 3.409091 |
| M. peachi | ABDUG Pal.D91 | 4 | ? | 4 | 3.466667 |
| M. peachi | ABDUG Pal.JA4 | 2 | 3.5 | 3 | 3.5 |
| M. peachi | ABDUG Pal.D56 | 7 | 5 | 4 | 4 |
| M. peachi | ABDUG Pal.JA1 | 3 | 3 | 4 | 4 |
| M. peachi | ABDUG Pal.D93b | ? | ? | ? | 4 |
| M. peachi | ABDUG Pal.D90 | 1.5 | 3.5 | 3.5 | 4.041096 |
| M. peachi | ABDUG Pal.D93i | 1 | 6 | 3 | 4.166667 |
| M. peachi | ABDUG Pal.D51 | ? | ? | ? | 4.333333 |
| M. peachi | ABDUG Pal.D214 | ? | ? | ? | 4.352941 |
|  |  |  |  |  |  |
| M. pusillus | ABDUG Pal.D54 | ? | ? | ? | 4.5 |
| M. pusillus | ABDUG Pal.D13 | ? | 2.5 | 2 | 4.666667 |
| M. pusillus | ABDUG Pal. D55 | 4 | 5 | 3.5 | 4.75 |
| M. pusillus | ABDUG Pal.JA2 | 2 | 4 | 3 | 4.761905 |
| M. pusillus | ABDUG Pal.D52 | 3.5 | 4 | 5 | 4.8125 |
| M. pusillus | ABDUG Pal.JA3 | 2 | 3 | 3 | 4.923077 |
| M. pusillus | ABDUG Pal.D53 | ? | ? | ? | 4.944444 |
| M. pusillus | GLAHM V3573 | ? | ? | ? | 5.015385 |
| M. pusillus | NHMUK PV P1329a | ? | ? | ? | 4.84375 |
| M. pusillus | NHMUK PV P1329b | ? | ? | ? | 4.75 |
| M. pusillus | GLAHM V2361 | ? | ? | ? | 5.652174 |
| M. pusillus | NHMUK PV P3578b | ? | ? | ? | 4.691057 |
| M. pusillus | NHMUK PV P9771 | ? | ? | ? | 6.253521 |
| M. pusillus | NHMUK PV P35784 | 3.7 | ? | 2.5 | 6.428571 |
| M. pusillus | NHMUK PV P40077 | ? | ? | ? | 4.1 |
| M. pusillus | NHMUK PV P40078 | ? | ? | ? | 5.890909 |
| M. pusillus | NHMUK PV P43019 | ? | 3.7 | 3.2 | 6.096154 |
|  |  |  |  |  |  |
| M. mitchelli | ABDUG Pal.D171 | 6 | 4 | 4 | 6.25 |
| M. mitchelli | ABDUG Pal.D172 | 5 | 6 | 7.5 | 6.666667 |
| M. mitchelli | ABDUG Pal.D173 | 5 | 7 | 5 | 5.318182 |
| M. mitchelli | NHMUK PV P1331 | 5.6 | 5.1 | 5.1 | 6.206186 |
| M. mitchelli | ABDUG Pal.12331a | ? | ? | ? | 6.533333 |
| M. mitchelli | ABDUG Pal.12229 | 7 | 7 | 8 | 7.111111 |
| M. mitchelli | ABDUG Pal.12236a | ? | ? | ? | 6 |
| M. mitchelli | ABDUG Pal.12236b | ? | ? | ? | 5.482759 |
| M. mitchelli | ABDUG Pal.12236c | ? | ? | ? | 7.333333 |
| M. mitchelli | ABDUG Pal.12236d | ? | ? | ? | 7.555556 |
| M. mitchelli | ABDUG Pal.12236f | ? | ? | ? | 5.5 |
| M. mitchelli | ABDUG Pal.12236h | ? | ? | ? | 7 |
| M. mitchelli | ABDUG Pal.12236i | ? | ? | ? | 4.8 |
| M. mitchelli | NMS G. 3053 ^ | 5 | 6.5 | 5 | 5.230769 |
| M. mitchelli | NMS G. 160 ^ | 4.5 | 5.5 | 5.5 | 6.1 |
| M. mitchelli | NMS G. 3054 ^ | ? | 5 | 7 | 5.777778 |
| M. mitchelli | NMS G. 46. 5 ^ | ? | ? | ? | 7.714286 |
| M. mitchelli | GLAHM V8118 b | ? | ? | ? | 5.017544 |
| M. mitchelli | GLAHM V8118 c | ? | ? | ? | 5.974684 |
| M. mitchelli | GLAHM V8118 d | ? | ? | ? | 4.824561 |
| M. mitchelli | NHMUK PV P9204a | ? | ? | ? | 6.300971 |
| M. mitchelli | NHMUK PV P4005 | 6.1 | 7.1 | 5.1 | 6.26087 |
| M. mitchelli | NMS G. 62 2 ^ | 6 | 8 | 9 | 5.631579 |
| M. mitchelli | NMS G. 62 3 ^ | 3 | 7.5 | 8.5 | 5 |
| M. mitchelli | NMS G. 62 4 ^ | 4 | ? | 8.5 | 5 |
| M. mitchelli | GLAHM V3522 | 6.2 | 6.5 | 9.2 | 5.732759 |
| M. mitchelli | GLAHM V3524 | 7.8 | ? | 4.6 | 4.771429 |
| M. mitchelli | NMS G. 1891 92. 306 | 6 | 8 | 5 | 5.166667 |
| M. mitchelli | NMS G. 1891 92. 301 | 5 | 7 | 9 | 7.2 |
| M. mitchelli | NMS G. 1891 92. 300 | 4 | 5 | 4 | 6.857143 |
| M. mitchelli | NMS G. 1891 92. 299 | ? | 5 | 8 | 4.866667 |
| M. mitchelli | NMS G. 1891 92. 309 | 4 | 4 | 4 | 5.444444 |
| M. mitchelli | NHMUK PV P560* | 5.8 | 4.8 | 6.4 | 6.3625 |
| M. mitchelli | NHMUK PV P1330 | 6.3 | 6.1 | 6.9 | 5.297297 |
| M. mitchelli | NHMUK PV P4004 | 3.6 | 5 | 6.4 | 5.671642 |
| M. mitchelli | NMS G. 1887 35. 1F | ? | ? | 9 | 5.916667 |
| M. mitchelli | NMS G. 1890 6. 28 | ? | ? | ? | 6.375 |
| M. mitchelli | NMS G. 1891 92. 276 | ? | 5 | 4 | 6.235294 |
| M. mitchelli | NMS G. 1891 92. 278 | 4 | 3.5 | 6 | 5 |
| M. mitchelli | NMS G. 1887 35. 1B | 8.5 | 8 | 8 | 5 |
| M. mitchelli | NMS G. 1891 92. 283 | 5.5 | 9 | 12 | 5.7 |
| M. mitchelli | NMS G. 1891 92. 287 | ? | ? | ? | 6.777778 |
| M. mitchelli | NMS G. 1891 92. 286 | 3 | 4 | 5 | 5.625 |
| M. mitchelli | NMS G. 1891 92. 281 | 5 | 7 | 9.5 | 4.857143 |
| M. mitchelli | NMS G. 1891 92. 285 | 4 | 5 | 7 | 5 |
| M. mitchelli | NMS G. 1891 92. 291 | 2.5 | 4 | 4 | 6.25 |
| M. mitchelli | NMS G. 1891 92. 280 | 3 | 5 | 6 | 5.857143 |
| M. mitchelli | GLAHM V3521 | ? | ? | ? | 6.153846 |
|  |  |  |  |  |  |
|  |  |  |  |  |  |
| Cheiracanthus sp. | ABDUG Pal.D68 | 25 | 30 | 40 | 4.375 |
| Cheiracanthus sp. | ABDUG Pal.D166 | 11 | 17 | 20 | 2.567568 |
| Cheiracanthus sp. | ABDUG Pal.D209 | 12 | 12 | 11 | 3.846154 |
| Cheiracanthus sp. | ABDUG Pal.D210 | 11 | 8 | 16 | 4 |
| Cheiracanthus sp. | ABDUG Pal.D211 | 9 | 12 | 19 | 3.642857 |
| Cheiracanthus sp. | ABDUG Pal.D212 | 8 | 11 | 11 | 4.52381 |

| **Taxon** | **Specimen number** | **L/LA** | **(PP+PA)/L** | **L/LD** | **LD/LD** |
| --- | --- | --- | --- | --- | --- |
| M. peachi | ABDUG Pal.10885 | ? | ? | ? | ? |
| M. peachi | ABDUG Pal.10886 | ? | ? | ? | ? |
| M. peachi | GLAHM V7019a | 1.506024 | 0.396 | 1.262626 | 1.192771 |
| M. peachi | GLAHM V7019b | 2.096 | ? | 1.969925 | 1.064 |
| M. peachi | GLAHM V7396 | ? | ? | ? | ? |
| M. peachi | GLAHM V7418 | 2.776596 | ? | 2.175 | 1.276596 |
| M. peachi | ABDUG Pal.10887 | ? | ? | ? | ? |
| M. peachi | NHMUK PV P61714 | 1.71875 | 0.290909091 | 1.624473 | 1.058036 |
| M. peachi | NHMUK PV P22201 | ? | ? | ? | ? |
| M. peachi | NHMUK PV P38583 | ? | ? | 1.947761 | ? |
| M. peachi | NHMUK PV P43967 | 1.914027 | 0.468085106 | 1.692 | 1.131222 |
| M. peachi | NHMUK PV P49668 | 1.714932 | 0.350923483 | 1.612766 | 1.063348 |
| M. peachi | NHMUK PV P49669 | 1.762712 | 0.34375 | 1.657371 | 1.063559 |
| M. peachi | NHMUK PV P61673 | 1.993151 | ? | 1.807453 | 1.10274 |
| M. peachi | NHMUK PV P61674 | ? | ? | ? | ? |
| M. peachi | NHMUK PV P61679a | 1.819048 | 0.403141361 | 1.43609 | 1.266667 |
| M. peachi | NHMUK PV P61679b | 2.048077 |  | 1.760331 | 1.163462 |
| M. peachi | NHMUK PV P61680 | 1.824427 | 0.380753138 | 1.731884 | 1.053435 |
| M. peachi | NHMUK PV P61682 | 2.03937 | 0.405405405 | ? | ? |
| M. peachi | NHMUK PV P61683 | ? | ? | 1.662983 | ? |
| M. peachi | NHMUK PV P61699 | ? | ? | ? | ? |
| M. peachi | NHMUK PV P61700 | 1.393103 | ? | 1.337748 | 1.041379 |
| M. peachi | NHMUK PV P61701 | ? | ? | ? | ? |
| M. peachi | NHMUK PV P61704 | 1.943662 | ? | 1.769231 | 1.098592 |
| M. peachi | NHMUK PV P61705 | ? | ? | ? | ? |
| M. peachi | NHMUK PV P61714 | 2.044586 | 0.3894081 | 1.969325 | 1.038217 |
| M. peachi | NHMUK PV P61715 | 1.779116 | 0.424379233 | 1.717054 | 1.036145 |
| M. peachi | NHMUK PV P61744 | 1.156028 | ? | 1 | 1.156028 |
| M. peachi | NHMUK PV P61766 | 1.885057 | ? | 1.757143 | 1.072797 |
| M. peachi | NHMUK PV P61768 | 1.771429 | ? | 1.734266 | 1.021429 |
| M. peachi | ABDUG Pal.D14 | ? | ? | 1.470588 | ? |
| M. peachi | ABDUG Pal.D92 | 1.764706 | ? | 1.578947 | 1.117647 |
| M. peachi | ABDUG Pal.D91 | 1.625 | 0.5 | 1.507246 | 1.078125 |
| M. peachi | ABDUG Pal.JA4 | 1.458333 | 0.428571429 | 1.4 | 1.041667 |
| M. peachi | ABDUG Pal.D56 | 1.694915 | 0.37 | 1.5625 | 1.084746 |
| M. peachi | ABDUG Pal.JA1 | 1.806452 | 0.428571429 | 1.6 | 1.129032 |
| M. peachi | ABDUG Pal.D93b | 1.882353 | 0.265625 | 1.684211 | 1.117647 |
| M. peachi | ABDUG Pal.D90 | 1.966667 | 0.338983051 | 1.735294 | 1.133333 |
| M. peachi | ABDUG Pal.D93i | 1.944444 | 0.371428571 | 1.842105 | 1.055556 |
| M. peachi | ABDUG Pal.D51 | 2.26087 | 0.442307692 | 2 | 1.130435 |
| M. peachi | ABDUG Pal.D214 | ? | ? | ? | ? |
|  |  |  |  |  |  |
| M. pusillus | ABDUG Pal.D54 | 2 | 0.37037037 | 1.741935 | 1.148148 |
| M. pusillus | ABDUG Pal.D13 | 1.555556 | 0.446428571 | 1.513514 | 1.027778 |
| M. pusillus | ABDUG Pal. D55 | 1.628571 | 0.421052632 | 1.540541 | 1.057143 |
| M. pusillus | ABDUG Pal.JA2 | 1.875 | 0.35 | 1.818182 | 1.03125 |
| M. pusillus | ABDUG Pal.D52 | 1.925 | 0.363636364 | 2.026316 | 0.95 |
| M. pusillus | ABDUG Pal.JA3 | 2.133333 | 0.34375 | 1.882353 | 1.133333 |
| M. pusillus | ABDUG Pal.D53 | ? | ? | ? | ? |
| M. pusillus | GLAHM V3573 | 1.63 | 0.371165644 | 1.559809 | 1.045 |
| M. pusillus | NHMUK PV P1329a | ? | ? | ? | ? |
| M. pusillus | NHMUK PV P1329b | ? | ? | ? | ? |
| M. pusillus | GLAHM V2361 | ? | ? | 1.733333 | ? |
| M. pusillus | NHMUK PV P3578b | 1.329493 | 0.428076256 | ? | ? |
| M. pusillus | NHMUK PV P9771 | ? | ? | ? | ? |
| M. pusillus | NHMUK PV P35784 | 1.679104 | 0.351111111 | 1.584507 | 1.059701 |
| M. pusillus | NHMUK PV P40077 | 1.877863 | ? | 1.744681 | 1.076336 |
| M. pusillus | NHMUK PV P40078 | ? | ? | ? | ? |
| M. pusillus | NHMUK PV P43019 | 1.713514 | ? | 1.695187 | 1.010811 |
|  |  |  |  |  |  |
| M. mitchelli | ABDUG Pal.D171 | 1.829268 | 0.36 | 1.704545 | 1.073171 |
| M. mitchelli | ABDUG Pal.D172 | 1.73913 | 0.35 | 1.6 | 1.086957 |
| M. mitchelli | ABDUG Pal.D173 | 2.207547 | 0.35042735 | 1.983051 | 1.113208 |
| M. mitchelli | NHMUK PV P1331 | 2.165468 | 0.264119601 | 1.905063 | 1.136691 |
| M. mitchelli | ABDUG Pal.12331a | ? | ? | ? | ? |
| M. mitchelli | ABDUG Pal.12229 | 2 | 0.3359375 | 1.882353 | 1.0625 |
| M. mitchelli | ABDUG Pal.12236a | ? | ? | ? | ? |
| M. mitchelli | ABDUG Pal.12236b | ? | ? | ? | ? |
| M. mitchelli | ABDUG Pal.12236c | ? | ? | ? | ? |
| M. mitchelli | ABDUG Pal.12236d | ? | ? | ? | ? |
| M. mitchelli | ABDUG Pal.12236f | ? | ? | ? | ? |
| M. mitchelli | ABDUG Pal.12236h | ? | ? | ? | ? |
| M. mitchelli | ABDUG Pal.12236i | ? | ? | ? | ? |
| M. mitchelli | NMS G. 3053 ^ | 2.428571 | 0.242647059 | 2.060606 | 1.178571 |
| M. mitchelli | NMS G. 160 ^ | 2 | 0.336065574 | 1.794118 | 1.114754 |
| M. mitchelli | NMS G. 3054 ^ | 1.925926 | 0.298076923 | 1.857143 | 1.037037 |
| M. mitchelli | NMS G. 46. 5 ^ | 1.862069 | 0.333333333 | 1.8 | 1.034483 |
| M. mitchelli | GLAHM V8118 b | ? | ? | 1.597765 | ? |
| M. mitchelli | GLAHM V8118 c | 1.942387 | 0.347457627 | 1.836576 | 1.057613 |
| M. mitchelli | GLAHM V8118 d | 1.833333 | 0.349090909 | 1.636905 | 1.12 |
| M. mitchelli | NHMUK PV P9204a | 1.707895 | 0.309707242 | 1.618454 | 1.055263 |
| M. mitchelli | NHMUK PV P4005 | 1.822785 | 0.369791667 | 1.75076 | 1.041139 |
| M. mitchelli | NMS G. 62 2 ^ | 1.981481 | 0.308411215 | 1.813559 | 1.092593 |
| M. mitchelli | NMS G. 62 3 ^ | 1.792453 | 0.368421053 | 1.637931 | 1.09434 |
| M. mitchelli | NMS G. 62 4 ^ | 1.549296 | 0.409090909 | 1.410256 | 1.098592 |
| M. mitchelli | GLAHM V3522 | 1.985075 | 0.284210526 | 1.837017 | 1.080597 |
| M. mitchelli | GLAHM V3524 | 2.0875 | 0.27994012 | 1.850416 | 1.128125 |
| M. mitchelli | NMS G. 1891 92. 306 | 1.86 | 0.387096774 | 1.722222 | 1.08 |
| M. mitchelli | NMS G. 1891 92. 301 | 2.25 | 0.333333333 | 2 | 1.125 |
| M. mitchelli | NMS G. 1891 92. 300 | 2.042553 | 0.375 | 1.882353 | 1.085106 |
| M. mitchelli | NMS G. 1891 92. 299 | 1.659091 |  | 1.586957 | 1.045455 |
| M. mitchelli | NMS G. 1891 92. 309 | 1.781818 | 0.357142857 | 1.53125 | 1.163636 |
| M. mitchelli | NHMUK PV P560* | 1.743151 | 0.400785855 | 1.626198 | 1.071918 |
| M. mitchelli | NHMUK PV P1330 | 1.689655 | 0.37244898 | 1.606557 | 1.051724 |
| M. mitchelli | NHMUK PV P4004 | 2.054054 | 0.486842105 | 1.784038 | 1.151351 |
| M. mitchelli | NMS G. 1887 35. 1F | 2.028571 | 0.281690141 | 1.868421 | 1.085714 |
| M. mitchelli | NMS G. 1890 6. 28 | ? | ? | ? | ? |
| M. mitchelli | NMS G. 1891 92. 276 | 2.038462 | 0.339622642 | 1.827586 | 1.115385 |
| M. mitchelli | NMS G. 1891 92. 278 | 1.774194 | 0.327272727 | 1.571429 | 1.129032 |
| M. mitchelli | NMS G. 1887 35. 1B | 1.886792 | 0.36 | 1.5625 | 1.207547 |
| M. mitchelli | NMS G. 1891 92. 283 | 2.035714 | 0.359649123 | 1.676471 | 1.214286 |
| M. mitchelli | NMS G. 1891 92. 287 | ? | ? | ? | ? |
| M. mitchelli | NMS G. 1891 92. 286 | 1.8 | 0.377777778 | 1.666667 | 1.08 |
| M. mitchelli | NMS G. 1891 92. 281 | 2.666667 | 0.382352941 | 2.385965 | 1.117647 |
| M. mitchelli | NMS G. 1891 92. 285 | 2.325581 | ? | 2 | 1.162791 |
| M. mitchelli | NMS G. 1891 92. 291 | 1.785714 | 0.38 | 1.5625 | 1.142857 |
| M. mitchelli | NMS G. 1891 92. 280 | 2.157895 | 0.268292683 | 1.863636 | 1.157895 |
| M. mitchelli | GLAHM V3521 | 1.693122 | 0.50625 | ? | ? |
|  |  |  |  |  |  |
|  |  |  |  |  |  |
| Cheiracanthus sp. | ABDUG Pal.D68 | 1.788321 | 0.342857143 | 2.378641 | 0.751825 |
| Cheiracanthus sp. | ABDUG Pal.D166 | 1.357143 | 0.642105263 | 1.727273 | 0.785714 |
| Cheiracanthus sp. | ABDUG Pal.D209 | 1.818182 | 0.31 | 1.960784 | 0.927273 |
| Cheiracanthus sp. | ABDUG Pal.D210 | 2.166667 | 0.346153846 | 2.810811 | 0.770833 |
| Cheiracanthus sp. | ABDUG Pal.D211 | 1.645161 | 0.5 | 1.728814 | 0.951613 |
| Cheiracanthus sp. | ABDUG Pal.D212 | 2.183908 | 0.352631579 | 2.567568 | 0.850575 |
